# Amphicarpic plants: definition, ecology, geographic distribution, systematics, life history, evolution and use in agriculture

**DOI:** 10.1111/brv.12623

**Published:** 2020-05-28

**Authors:** Keliang Zhang, Jerry M. Baskin, Carol C. Baskin, Gregory P. Cheplick, Xuejun Yang, Zhenying Huang

**Affiliations:** ^1^ State Key Laboratory of Vegetation and Environmental Change Institute of Botany, Chinese Academy of Sciences Beijing 100093 P.R. China; ^2^ Jiangsu Key Laboratory of Crop Genetics and Physiology, College of Horticulture and Plant Protection Yangzhou University Yangzhou 225009 P.R. China; ^3^ Department of Biology University of Kentucky Lexington KY 40506 USA; ^4^ Department of Plant and Soil Sciences University of Kentucky Lexington KY 40546 USA; ^5^ Department of Biology City University of New York Staten Island NY 10314 USA

**Keywords:** amphicarpy, bet‐hedging, chasmogamous and cleistogamous flowers, life history, phylogeny, reproductive plasticity

## Abstract

Although most plants produce all of their fruits (seeds) aboveground, amphicarpic species produce fruits (seeds) both above‐ and belowground. Our primary aims were to determine the number of reported amphicarpic species and their taxonomic, geographic, life form and phylogenetic distribution, to evaluate differences in the life history of plants derived from aerial and subterranean seeds, to discuss the ecological and evolutionary significance of amphicarpy, to explore the use of amphicarpic plants in agriculture, and to suggest future research directions for studies on amphicarpy. Amphicarpy occurs in at least 67 herbaceous species (31 in Fabaceae) in 39 genera and 13 families of angiosperms distributed in various geographical regions of the world and in various habitats. Seeds from aerial and subterranean fruits differ in size/mass, degree of dormancy, dispersal and ability to form a persistent seed bank, with aerial seeds generally being smaller, more dormant and more likely to be dispersed and to form a seed bank than subterranean seeds. In addition, plants produced by aerial and subterranean seeds may differ in survival and growth, competitive ability and biomass allocation to reproduction. Amphicarpic plants may exhibit a high degree of plasticity during reproduction. Subterranean fruits are usually formed earlier than aerial ones, and plants may produce only subterranean propagules under stressful environmental conditions. Differences in the life histories of plants from aerial and subterranean seeds may be an adaptive bet‐hedging strategy.

## I. INTRODUCTION

Sexual reproduction is a core component of many life‐history strategies, and it often serves as the focal point for studies on the evolutionary process (Silvertown & Charlesworth, 2001; Goodwillie, Kalisz, & Eckert, 2005). The persistence of a sexually reproducing species in its natural habitat depends on seed production and recruitment of new individuals to the population (Baskin & Baskin, 2014). Plant propagation by sexual reproduction can increase the number of individuals and expand the distribution range and increase the genetic variability of the species (Cocks, 1999; Silvertown & Charlesworth, 2001). In flowering plants, reproductive strategies and mating systems vary greatly among species and may involve outcrossing, selfing or some combination of the two (mixed mating) (Goodwillie *et al*., 2005).

In nature, most plants produce fruits and seeds only aboveground, but some species produce sexual propagules belowground. In the peanut (*Arachis hypogaea*), for example, the fertilized ovary of the sessile chasmogamous flower penetrates the soil by means of an elongating ‘peg’, the tip of which enlarges to form the subterranean pod (Darwin, 1888; Kaul, Koul, & Sharma, 2000). However, some species produce both aerial and subterranean fruits and seeds on the same plant, a phenomenon known as amphicarpy (Cheplick, 1987; Kaul *et al*., 2000; Barker, 2005; Kumar, Lawn, & Bielig, 2012; Baskin & Baskin, 2014; Koontz *et al*., 2017). This dual reproductive strategy maximizes fitness, since it combines the advantages of both types of seeds (Cheplick, 1987, 1994; Kaul *et al*., 2000).

In amphicarpic plants, aerial and subterranean seeds (fruits) differ in size/mass, dispersal ability and/or degree of dormancy. In addition, plants produced by aerial and subterranean seeds often differ in many ways including survival and growth, competitive ability and biomass allocation to reproduction (Table 1). Thus, amphicarpy can be considered a subset of a much larger set of plant species that exhibit reproductive dimorphism (Plitmann, 1995). Consequently, the selective factors responsible for the evolution of amphicarpy may be similar to those proposed to explain the evolution of reproductive dimorphism.

**Table 1:** Differences in aerial (A) and subterranean (S) seeds of amphicarpic plants and of the plants derived from them. CH, chasmogamous; CL, cleistogamous

Seed morphology/structure/physiology	
Desiccation sensitivity	A < S (Schnee & Waller, 1986; Zhang *et al*., 2015)
Fruits dehiscent *versus* indehiscent	A fruits dehiscent, S fruits indehiscent (Maheshwari & Maheshwari, 1955; Speroni & Izaguirre, 2001; Kumar *et al*., 2012; Zhang *et al*., 2015)
Moisture content	A < S (Schnee & Waller, 1986; Zhang *et al*., 2015)
Seed coat and pericarp anatomy	A seedcoat well developed, S seedcoat not well developed (Zhang *et al*., 2015)
Seed morphology/structures associated with diaspores	Differs among morphs and species (Alinoglu & Durlu, 1970; Durlu & Cornelius, 1970; Evenari *et al*., 1971; Weiss, 1980; Gopinathan & Babu, 1986; Ruiz de Clavijo, 1995; Ruiz de Clavijo & Jimenez, 1998; Conterato, Schifino‐Wittmann, & Dall'Agnol, 2010; Zhang *et al*., 2015)
Seed size/mass	A < S (Maheshwari & Maheshwari, 1955; Koller & Roth, 1964; Alinoglu & Durlu, 1970; Durlu & Cornelius, 1970; Evenari *et al*., 1971; Cheplick, 1983, 1987, 1994; Cheplick & Quinn, 1983; Gopinathan & Babu, 1986; Schnee & Waller, 1986; Trapp & Hendrix, 1988; Kawano *et al*., 1990; Ruiz de Clavijo, 1995; Ruiz de Clavijo & Jimenez, 1998; Conterato *et al*., 2010; Talavera *et al*., 2010; Kumar *et al*., 2012; Choo *et al*., 2014, 2015; Zhang *et al*., 2015)
Seed dispersal	
Dispersal ability	A > S (Koller & Roth, 1964; Evenari *et al*., 1971; Mattatia, 1977a,b; Ruiz de Clavijo, 1995; Ruiz de Clavijo & Jimenez, 1998; Talavera *et al*., 2010; Zhang *et al*., 2015)
Seedlings from A and S seeds	
Growth	A < S (Koller & Roth, 1964; Cheplick, 1988)
Size	A < S (Koller & Roth, 1964; Loria & Noy‐Meir, 1979/80; Weiss, 1980; Schnee & Waller, 1986; Cheplick, 1987; Trapp & Hendrix, 1988; Talavera *et al*., 2010; Choo *et al*., 2015)
Stress tolerance and competitive ability	A < S (Koller & Roth, 1964; Evenari *et al*., 1971; Loria & Noy‐Meir, 1979/80; Cheplick, 1987) A = S (Kim *et al*., 2016)
Survival	A < S (Loria & Noy‐Meir, 1979/80; Cheplick & Quinn, 1982; Cheplick, 1987, 1988; Talavera *et al*., 2010)
Vegetative growth of plants derived from A and S seeds	
Allocation of biomass to reproductive parts	Plants from S seeds allocate more biomass to A seed production than plants from A seeds (Cheplick, 1983, 1987; Cheplick & Quinn, 1983; Ruiz de Clavijo & Jimenez, 1998; Kaul *et al*., 2002; Choo *et al*., 2015) A = S (Kim *et al*., 2016)
Competitive ability	A < S (Weiss, 1980; Cheplick & Quinn, 1982, 1983; Ruiz de Clavijo & Jimenez, 1998; Sadeh *et al*., 2009; Zhang *et al*., 2017)
Leaf number/area	A < S (Weiss, 1980; Trapp & Hendrix, 1988; Choo *et al*., 2015; Zhang *et al*., 2017)
Plant size/dry matter production	A < S (Ruiz de Clavijo & Jimenez, 1998; Choo *et al*., 2015; Zhang *et al*., 2017) A = S (Kim *et al*., 2016)
Relative growth rate	A < S (Cheplick, 1983; Cheplick & Quinn, 1987, 1988*b*) A = S (Kim *et al*., 2016)
Root/shoot mass ratio	Root/shoot ratio of plants derived from A and S seeds were negatively affected by nutrient availability and positively affected by intraspecific density (Sadeh *et al*., 2009)

Reproduction	
Flower development	A and S flowers diverged at mid‐ to late development stage (Zhang *et al*., 2006) A and S flowers have normal ovule and embryo sac development (Speroni *et al*., 2010)
Flower size and number	A > S (Durlu & Cornelius, 1970; Fukui & Takahashi, 1975; Trapp & Hendrix, 1988; Jiang & Kadono, 2001; Zhang *et al*., 2006)
Flower structure/colour/anatomy	A CH flowers are large and brightly coloured and S flowers are small and white (Alinoglu & Durlu, 1970; Durlu & Cornelius, 1970; Gopinathan & Babu, 1986; Schnee & Waller, 1986; Trapp & Hendrix, 1988; Speroni & Izaguirre, 2001; Zhang *et al*., 2006; Conterato *et al*., 2010; Kumar *et al*., 2012) A and S flower size not influenced by position on the plant (Ortiz *et al*., 2009)
Flower types	A CH and CL and S CL (Schnee & Waller, 1986; Gopinathan & Babu, 1987; Trapp, 1988; Trapp & Hendrix, 1988; Zhang *et al*., 2005; Kawano, 2008) (amphicarpic *sensu stricto*) A CH and S CL (Maheshwari & Maheshwari, 1955; Raynal, 1967; Cheplick, 1983; Cheplick & Quinn, 1988*b*; Speroni & Izaguirre, 2001, 2003) (amphicarpic *sensu stricto*) CL are submerged and CH emerged in two aquatic macrophyte taxa of *Blyxa* (Jiang & Kadono, 2001) (amphicarpic *sensu stricto*) A CH and basal (but not subterranean) CH (Koller & Roth, 1964; Evenari *et al*., 1977; Weiss, 1980; Ruiz de Clavijo, 1995; Ruiz de Clavijo & Jimenez, 1998) (amphicarpic *sensu lato*)
Number of inflorescences	Plants derived from A < S (Alinoglu & Durlu, 1970; Ruiz de Clavijo & Jimenez, 1998; Kumar *et al*., 2012)
Phenotypic plasticity	Plants derived from A > S (Cheplick, 1983, 1994; Cheplick & Quinn, 1983; Kawano *et al*., 1990; Ruiz de Clavijo & Jimenez, 1998; Sadeh *et al*., 2009)
Pollen viability (%)	Plants derived from A = S (Conterato *et al*., 2013)
Pollen/ovule ratio	Plants derived from A > S (Gopinathan & Babu, 1986; Kaul *et al*., 2002; Kawano, 2008; Kumar *et al*., 2012)
Ratio of seed and fruit number to seed and fruit mass	Plants derived from A > S (Cheplick, 1987; Kawano *et al*., 1990; Sadeh *et al*., 2009; Conterato *et al*., 2010)
Reproductive output of plants derived from A and S seeds	Plants derived from A < S (McNamara & Quinn, 1977; Loria & Noy‐Meir, 1979/80; Cheplick & Quinn, 1982, 1988*b*; Cheplick, 1987, 1994; Trapp & Hendrix, 1988; Kawano *et al*., 1990; Jiang & Kadono, 2001; Conterato *et al*., 2010) Plants derived from A = S (Kim *et al*., 2016)
Seed number/plant	Plants derived from A > S (Maheshwari & Maheshwari, 1955; Fukui & Takahashi,1975; Cheplick, 1983, 1987, 1988; Gopinathan & Babu, 1986; Cheplick & Quinn, 1987; Ruiz de Clavijo, 1995; Choo *et al*., 2014; Nam *et al*., 2017)
Seed set (%)	S CL flowers > A CH flowers (McNamara & Quinn, 1977; Cheplick & Quinn, 1986; Jiang & Kadono, 2001; Speroni *et al*., 2010)
Time to flowering	Plants derived from A < S (Zeide, 1978; Cheplick & Quinn, 1982; Cheplick, 1983) S flowers open earlier than A flowers (Schnee & Waller, 1986; Ruiz de Clavijo, 1995; Ruiz de Clavijo & Jimenez, 1998; Berjano *et al*., 2014; Choo *et al*., 2014) A flowers open earlier than S flowers (Kim *et al*., 2016)
Life history	
Life history trade‐offs	A flower and seed production are correlated with vegetative mass (Zeide, 1978; Weiss, 1980; Schnee & Waller, 1986 ; Trapp & Hendrix, 1988), while S flower and fruit production are (Schnee & Waller, 1986; Trapp & Hendrix, 1988) or are not correlated (Zeide, 1978; Weiss, 1980)
Quantitative genetics of life‐history traits	Seed set and seed mass of both seed types had low quantitative genetic variation relative to other traits (Cheplick & Quinn, 1988*a*; Cheplick, 1994)

Dormancy and germination	
Degree (depth) of dormancy	A > S (Koller & Roth, 1964; Alinoglu & Durlu, 1970; McNamara & Quinn, 1977; Walker & Evenson, 1985*b*; Schnee & Waller, 1986; Trapp & Hendrix, 1988; Ruiz de Clavijo, 1995; Ruiz de Clavijo & Jimenez, 1998; Choo *et al*., 2014, 2015; Zhang *et al*., 2015) A < S (Evenari *et al*., 1977)
Germination and viability response to storage	Germination of A and S decreased with dry storage in *Amphicarpum amphicarpon* (McNamara & Quinn, 1977) Germination of A increased with dry storage, whereas S lost viability (Zhang *et al*., 2015)
Germination of water‐permeable seeds	Scarification of seed coat increased germination of A more than it did in S (Walker & Evenson, 1985*b*)
Germination percentage	A > S (Koller & Roth, 1964; Alinoglu & Durlu, 1970; Durlu & Cornelius, 1970; Weiss, 1980; Ruiz de Clavijo, 1995) A < S (McNamara & Quinn, 1977; Gamm, 1983; Walker & Evenson, 1985*b*; Schnee & Waller, 1986; Trapp & Hendrix, 1988; Choo *et al*., 2015; Zhang *et al*., 2015)
Germination response to cold stratification	Cold stratification increased germination of intact A and S seeds (McNamara & Quinn, 1977) Cold stratification increased germination percentage of scarified A seeds and of intact S seeds (Zhang *et al*., 2015)
Germination response to dry heat	Dry heat increased germination of A seeds more than it did for S (Walker & Evenson, 1985*b*)
Germination response to light	Light increased germination of A seeds more than it did for S seeds (Weiss, 1980) Light increased germination of S seeds more than it did for A seeds (Walker & Evenson, 1985*b*) Light increased germination of both A and S seeds (Koller & Roth, 1964) Light decreased germination of both A and S seeds (Ruiz de Clavijo, 1995) Light had no effect on germination of either A or S seeds (Zhang *et al*., 2015)
Germination response to temperature	Subterranean seeds usually germinate to a higher percentage than those of A at a given temperature (Koller & Roth, 1964; McNamara & Quinn, 1977; Weiss, 1980; Walker & Evenson, 1985*b*; Choo *et al*., 2015; Zhang *et al*., 2015; Baskin & Baskin, 2017)
Permeability to water and storage behaviour	Aerial seeds are water impermeable and orthodox and S water permeable and recalcitrant (Schnee & Waller, 1986; Zhang *et al*., 2015)
Germination ecology	
Ability to form a persistent seed bank	A > S (Cheplick, 1987; Zhang *et al*., 2015)
Soil burial depth of seedling emergence	Greater seed burial depth decreased emergence percentages of A and S. With increase of seed burial depth, plants reared from both A and S seeds allocated more biomass to S seeds (Walker & Evenson, 1985*a*; Cheplick & Quinn, 1987)
Seed germination phenology	Usually no difference in timing of seed germination (Cheplick, 1987) Earlier germination of S seeds (Choo *et al*., 2015)
Effect of abiotic and biotic environment on proportion of the two morphs	
Competition (density)	Plants from A seeds had significantly less total growth and seed production than those from S seeds in both high and low competition, and S seed production was less affected than A seed production by density in both A and S plants (Weiss, 1980; Cheplick & Quinn, 1982, 1983; Ruiz de Clavijo & Jimenez, 1998; Cheplick, 2007; Sadeh *et al*., 2009; Nam *et al*., 2017)
Fire	S seeds (protected from fire) germinated after the fire, whereas A seeds were killed by fire (Cheplick & Quinn, 1987, 1988*b*)
Flooding	Flooding damage during late vegetative growth tended to decrease production of S seeds and total plant mass, while allocation to A seeds did not differ among treatments (Choo *et al*., 2014)
Pathogens	Infection by fungi increased pre‐reproductive mortality and decreased seed production of small and large plants of *Amphicarpaea bracteata*, whereas smaller plants were more affected. With an increase in intensity of fungal infection, the proportion of CL seeds (presumably) increased (Parker, 1986)
Irradiance	Plants from A seeds had significantly less total growth and seed production than those from S seeds under the same irradiance. However, with an increase in irradiance the number of A seeds increased more than that of S seeds for plants from both A and S seeds (Trapp & Hendrix, 1988; Berjano *et al*., 2014; Nam *et al*., 2017; Zhang *et al*., 2017)
Litter	S seeds on bare soil surface in clay pots were more likely to lose viability and less likely to germinate than seeds protected by litter or by burial in soil (Cheplick & Quinn, 1987)
Reciprocal transplanting between populations	S seeds placed in the same habitat as the parents produced seedlings of greater vigour and adults of higher reproductive capacity than plants from seeds transplanted to a different habitat far removed from the parents (Cheplick, 1988)
Soil moisture	Plants from A seeds had significantly less total growth and seed production than those from S seeds at both dry and wet sites (Cheplick & Quinn, 1982)
Substrate fertility (nutrients)	Plants from A seeds had significantly less total growth and seed production than those from S seeds under the same nutrient availability (Weiss, 1980; Cheplick, 1987, 1989; Jiang & Kadono, 2001; Sadeh *et al*., 2009; Kim *et al*., 2016) With an increase of nutrient availability, total A/S seed mass of plants derived from both A and S plants increased (Weiss, 1980; Cheplick, 1987, 1989; Sadeh *et al*., 2009; Kim *et al*., 2016)

[Correction added on 26 June 2020, after online publication: Table headings for Table 1 have been amended in this current version.]

Research by various authors has contributed greatly to our understanding of the differences between aerial and subterranean seeds and the life‐history performance of the plants that arise from them. However, the definition of amphicarpy is complex, and its ecological and evolutionary significance has received relatively little attention. The aims of this review are to (*i*) clarify the definition of amphicarpy; (*ii*) survey the reported occurrence of amphicarpy in angiosperm families, genera and species; (*iii*) determine the geographical distribution, habitats and life forms of amphicarpic species; (*iv*) review the differences in life‐history traits of plants derived from aerial and subterranean seeds; (*v*) discuss the ecological consequences and evolution of amphicarpy; (*vi*) determine the use of amphicarpic legumes in agriculture; and (*vii*) suggest future research approaches on amphicarpic species.

## II. DEFINITION OF AMPHICARPY

**Fig 1:**
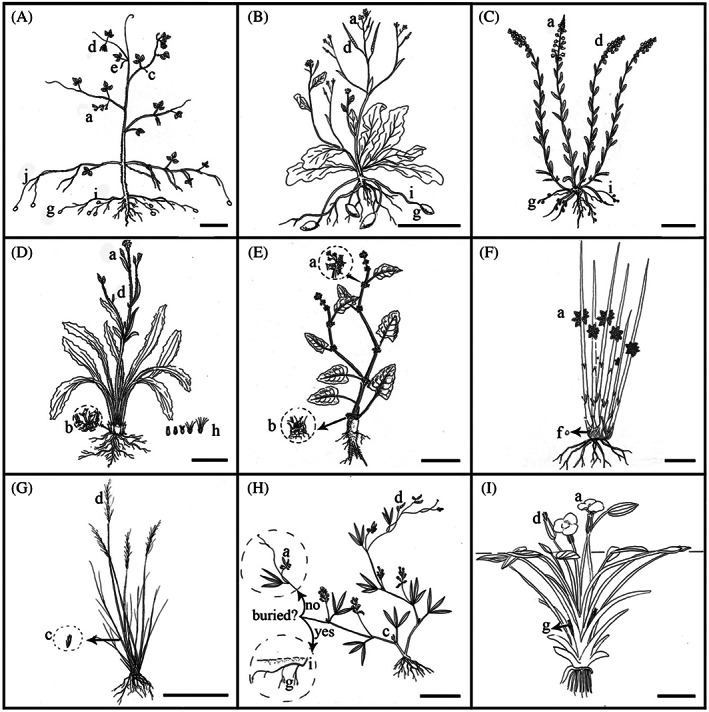
Drawings of plants of (A) *Amphicarpaea edgeworthii*; (B) *Cardamine chenopodifolia*; (C) *Polygala polygama*; (D) *Catananche lutea*; (E) *Emex spinosa*; (F) *Schoenoplectiella articulata*; (G) *Achnatherum caudatum*; (H) *Glycine pindanica*; and (I) *Ottelia ovalifolia*. a, aerial chasmogamous flowers; b, ground‐level chasmogamous flowers; c, aerial cleistogamous flowers; d, aerial chasmogamous fruits; e, aerial cleistogamous fruits; f, ground‐level fruits (basicarps); g, subterranean fruits; h, subterranean and aerial achenes of *Catananche lutea* (the five fruits of *C. lutea* from left to right are amphic‐1 and amphic‐2 produced by ground‐level chasmogamous flowers, and peripheral, intermediate and central fruits produced by aerial flowers); i, subterranean stem; j, aerial axillary shoot that arises from the first node that becomes buried in soil. All drawings are by K. Zhang: A modified from Zhang *et al*. (2015); B modified from Cheplick (1987); C and D modified from Plant illustrations website (http://www.Plantillustrations.org); fruits in D modified from Ruiz de Clavijo (1995); E modified from Ortiz *et al*. (2009); F modified from Lye (2003); G modified from Plant illustrations website based on the description by Barker (2005); H based on the description by Tindale & Craven (1993) and various online photographs; I modified from Plant illustrations website based on the description by Ernst‐Schwarzenbach (1956). Scale bars = 5 cm.

The word ‘amphicarpy’ is derived from the combination of the Greek words *amphi* (both or around) and *carpos* (fruits); nothing about the term implies that any fruits/seeds are belowground. However, the use of amphicarpy differs among researchers. Haines (1971) and Bruhl (1994) defined amphicarpy as the production of aerial fruits and fruits at ground level or belowground on the same plant (Fig. 1A–F). van der Pijl (1982) and Cheplick (1987) used amphicarpy for plants that produce aerial and subterranean fruits (Fig. 1A–E). Barker (2005) divided amphicarpy into aerial amphicarpy, amphi‐geocarpy and amphi‐basicarpy. Aerial amphicarpy refers to different kinds of flowers and fruits produced aboveground (Fig. 1G), amphi‐geocarpy to flowers and fruits produced above‐ and belowground (Fig. 1A–E) and amphi‐basicarpy to flowers and fruits produced aboveground and at ground level (Fig. 1F). Baskin & Baskin (2014) divided amphicarpy into amphicarpy *sensu stricto* and amphicarpy *sensu lato*. The former refers to plants that produce subterranean and aerial flowers and fruits (Fig. 1A–C) and the latter to plants that produce aerial flowers and fruits and flowers near the soil surface that are pulled underground and produce subterranean fruits (Fig. 1D, E). In amphicarpic *sensu stricto* species, plants produce aerial chasmogamous (CH), or aerial CH and cleistogamous (CL), flowers and subterranean CL flowers. In amphicarpy *sensu lato* species, plants produce aerial CH, or aerial CH and CL, flowers and CH flowers near the soil surface aboveground (not subterranean).

Most amphicarpic species routinely produce both aerial and subterranean seeds. However, in some species, subterranean seeds are produced only in certain circumstances (Tindale & Craven, 1988; Kollipara, Singh, & Hymowitz, 1997). Tindale & Craven (1988) distinguished between ‘habitual’ amphicarpy and ‘opportunistic’ amphicarpy. Subterranean seeds are regularly produced in habitual amphicarpy (Fig. 1A–E), while species with opportunistic amphicarpy have prostrate stems and under certain circumstances (for example, when stems are covered by soil or leaf litter) they produce colourless branches that bear CL flowers (Fig. 1H) (Tindale & Craven, 1988; Kollipara *et al*., 1997). Opportunistic amphicarpy, is commonly found in *Glycine arenaria*, *G. hirticaulis*, *G. pindanica*, *G. pullenii* and *G. tomentella* (Tindale & Craven, 1993; Pfeil & Craven, 2002).

Herein, we define amphicarpy as the production of above‐ and belowground fruits [amphi‐geocarpy of Barker (2005) includes both amphicarpy *sensu stricto* and amphicarpy *sensu lato* of Baskin & Baskin (2014)]. Fruits produced near ground level, such as in some *Bulbostylis* and *Schoenoplectiella* species of Cyperaceae (Haines, 1971; Bruhl, 1994) and *Ceratocarpus arenarius* (Amaranthaceae) (Lu *et al*., 2013) are not amphicarpic but amphi‐basicarpic. In the aquatic species *Blyxa aubertii* var*. aubertii*, *B. aubertii* var. *echinosperma* and *Ottelia ovalifolia* (Fig. 1I), flowers and fruits are produced below the surface of the water and other flowers and fruits above the water surface (Ernst‐Schwarzenbach, 1956; Jiang & Kadono, 2001); this pattern of sexual reproduction also is considered to be amphicarpy. Species with opportunistic amphicarpy were not included in our list of amphicarpic species.

## III. SYSTEMATIC/PHYLOGENETIC OCCURRENCE AND LIFE FORM

To identify amphicarpic species, an extensive literature search was conducted using online databases. We defined a species as amphicarpic (or not) based on the descriptions and illustrations (where available) of each amphicarpic species in the literature. A few cases of purported amphicarpy in the literature only provided a name, and we searched for the original reference. If the description in the original reference was vague, we verified the presence of amphicarpy by using online search engines, various regional floras and virtual herbaria to locate additional information on the species in question. Amphicarpic species of *Clitoria* sp., *Neocracca* sp., *Orobus*, *Eremiti*, and *Libyella cyrenaica* were difficult to verify; nevertheless, we counted these taxa as being amphicarpic. Plants of *Pisum fulvum* (Fabaceae) exhibit a gradient from both aerial and subterranean flowers and fruits (amphicarpic plants *sensu stricto*) to plants with only aerial flowers and fruits (Mattatia, 1977*a*). Also, some plants are basicarpic with CH flowers near the soil surface, i.e. ‘sub‐amphicarpic’ (Mattatia, 1977*a*). We included *P. fulvum* in our list of amphicarpic species (but see footnote d to Table S1). To avoid synonymy, we checked the name of each species using the *The Plant List* (2013; http://www.theplantlist.org). The nomenclature of species, genera and families was updated to reflect currently accepted names (see online Supporting information, Table S1).

We found 108 species in the literature reported to be ‘amphicarpic’, of which 36 belonging to six genera and three families [Amaranthaceae (1 genus:1 species); Cyperaceae (4:34) and Poaceae (1:1)] are amphi‐basicarpic. Five species of *Glycine* had opportunistic amphicarpy (Fig. 1H). Sixty‐seven of the 108 species in 39 genera and 13 families (Table 2; Table S1) are amphicarpic, i.e. fruits are produced both above‐ and belowground. Three families have more than five species each: Fabaceae (15 genera:31 species), Poaceae (6:11) and Commelinaceae (3:6). Amphicarpy has been reported in five tribes in Fabaceae: Phaseoleae (8:18) (*Amphicarpaea*, *Centrosema*, *Clitoria*, *Flemingia*, *Galactia*, *Glycine*, *Macroptilium* and *Vigna*); Vicieae (4:9) (*Orobus*, *Pisum*, *Lathyrus* and *Vicia*); Trifolieae (1:2) (*Trifolium*); Robinieae (1:1) (*Neocracca*); and Tephrosieae (1:1) (*Tephrosia*).

**Table 2:** Taxonomic distribution of amphicarpic species (see complete list in Table S1)

Families	No. genera	No. species
Asteraceae	2	2
Brassicaceae	2	2
Commelinaceae	3	6
Cyperaceae	1	1
Fabaceae	15	31
Gentianaceae	1	1
Hydrocharitaceae	2	3
Poaceae	6	11
Polygalaceae	1	3
Polygonaceae	3	4
Scrophulariaceae	1	1
Urticaceae	1	1
Violaceae	1	1
Total	39	67

**Fig 2:**
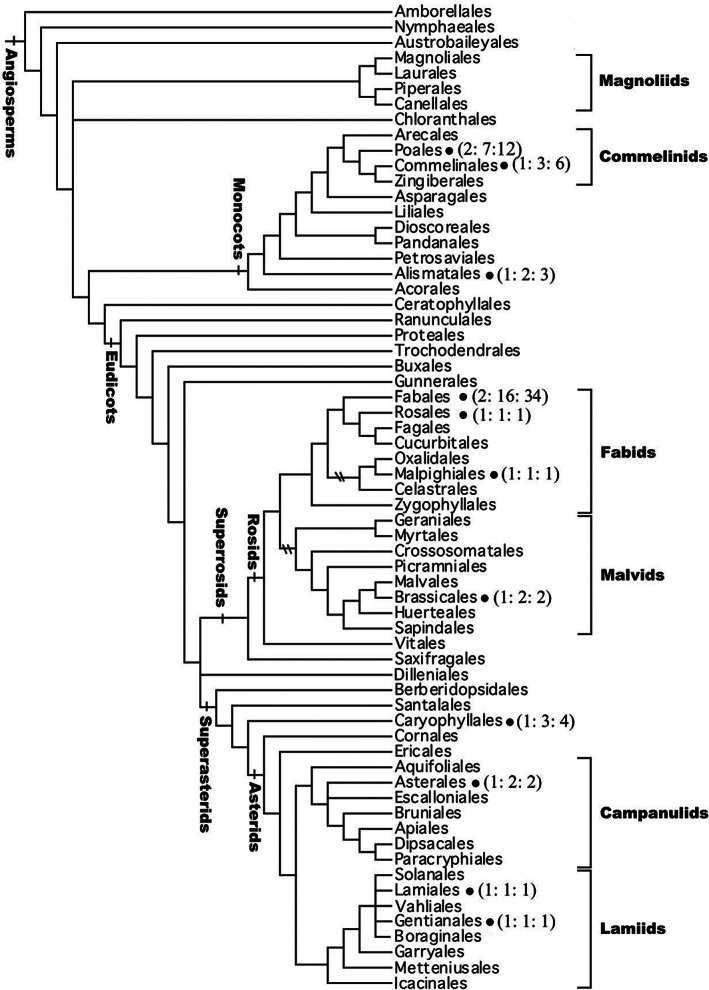
Ordinal phylogenetic position of amphicarpic species. (X: X: X) represents number of families, genera and species in orders in which amphicarpic species have been documented. The phylogenetic diagram is modified from the Angiosperm Phylogeny Group (2016).

The phylogenetic position of orders that contain plant families with amphicarpic species shows that amphicarpy is found mainly in phylogenetically advanced families (Fig. 2). No families of the ANA grade (Amborellales, Nymphaeles, Austrobaileyales) or magnoliids contain species reported to be amphicarpic. Four families of monocots and nine of eudicots are reported to be amphicarpic; 31 of the 67 species are in the Fabaceae (Table 2; Table S1). The phylogenetically widespread distribution of amphicarpy within angiosperms suggests that this reproductive strategy has evolved repeatedly.

Life cycle and life form were determined for 59 species. All 59 species are herbs: 28 (47.5%) are annuals, 28 (47.5%) perennials and three (5%) are annuals or perennials. *Clitoria*, *Neocracca* and *Orobus* were tabulated as one species each and *Eremitis* as five species, which were not named in our sources; thus, type of life cycle and life form were not determined for eight species. With respect to Raunkiaer life forms for the 59 species, 28 (47.5%) are therophytes (annuals), 10 (17%) hemicryptophytes, seven (12%) geophytes, eight (14%) chamaephytes, three (5%) hydrotherophytes or hydrohemicryptophytes and three (5%) therophytes or hemicryptophytes.

## IV. GEOGRAPHICAL DISTRIBUTION AND ADAPTATION TO HABITAT

Amphicarpy generally has been considered to be an adaptation to dry habitats (Zohary, 1937; van der Pijl, 1982; Cheplick, 1987). For example, Zohary (1937) listed about 30 species (mainly Fabaceae) with amphicarpy occurring worldwide in arid regions. Plitmann (1973) listed 10 amphicarpic species in the Israel flora, of which three (*Catananche lutea*, *Gymnarrhena micrantha* and *Emex spinosa*) are amphicarpic *sensu lato* and seven amphicarpic *sensu stricto*. Cheplick (1987) reported 29 amphicarpic species and noted that ‘with a few notable exceptions, they inhabit dry habitats such as deserts’ (Cheplick, 1987, p. 97). Examples of amphicarpic species distributed in arid and desert regions include *Emex spinosa* (Evenari, Kadouri, & Gutterman, 1977), *Gymnarrhena micrantha* (Koller & Roth, 1964), *Lathyrus amphicarpos* (Mattatia, 1977*b*; Cheplick, 1987), *L. ciliolatus* (Mattatia, 1977*b*), *L. hierosolymitanus* (Lev‐Yadun, 2000), *Pisum fulvum* (Mattatia, 1977*a*) and *Scrophularia arguta* (van der Pijl, 1982). In the Negev desert of Israel, 78% of the subterranean seeds of *Emex spinosa* emerged from 1–4 cm soil depth, while no aerial seeds germinated on the soil surface (Evenari *et al*., 1977). Moreover, in drought years, only a few plants are derived from aerial seeds, while in wet years more aerial seeds formed and dispersed. As conditions vary remarkably among years, populations expand or contract around safe sites that are sufficiently wet (Evenari *et al*., 1977).

However, the occurrence of *Amphicarpaea bracteata* (Schnee & Waller, 1986) and *Commelina virginica* (Kaul *et al*., 2000) in eastern North America and *A. edgeworthii* in East Asia (Zhang *et al*., 2015) show that amphicarpy is not restricted to arid habitats. Plitmann (1986) also reported 55–58 amphicarpic species, among which 24 amphicarpic species grow in tropical or subtropical regions and 31 in temperate regions, 14 of which are in the Mediterranean region and only three or four in arid zones.

**Fig 3:**
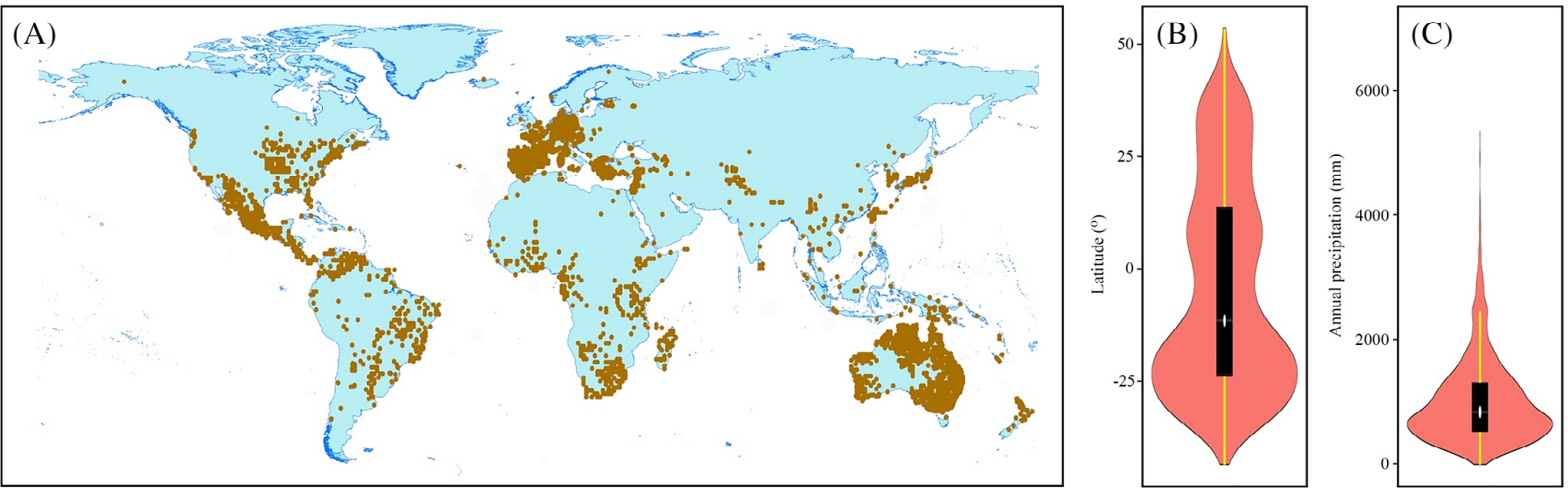
(A) Global distribution (orange dots) of amphicarpic species and (B, C) violin plots showing the density of records with respect to (B) latitude and (C) annual precipitation. The shape of B and C represents the density estimate of the variable (the more data points in a specific range, the larger the violin is for that range). The white dot in the black bars of B and C is the median value, the thick black bar the interquartile range and thin line extending from the black bar the upper (max) and lower (min) adjacent values in the data.

We used geo‐referenced data obtained from Global Biodiversity Information Facility (GBIF; https://www.gbif.org) to estimate the geographic distribution of amphicarpic species. Raw data were examined by hand and with automated scripts for obvious mistakes such as collection sites that occurred in the sea or where the sign of the coordinates was inverted (Rubio de Casas *et al*., 2017). With the help of ArcGIS 10.2, a sighting point map was developed. Annual precipitation data obtained from the WorldClim global climate database (www.worldclim.org) were extracted based on species occurrence records and further analysed for their relationship with the presence of amphicarpy. We found that amphicarpic species are widely distributed and occur in temperate, subtropical, tropical and arid/semiarid regions (Table S1; Fig. 3A). The latitudinal range of amphicarpic species extends from 44.5° S to 68.5° N, with the greatest abundance of individuals and species at approximately 25° S (Fig. 3B). Amphicarpic species occur where annual precipitation ranges from 10 to 5300 mm, and they are most abundant at around 1000 mm (Fig. 3C). Therefore, we can conclude that amphicarpic species are not most abundant in desert regions.

In some amphicarpic species in temperate regions, amphicarpy has been viewed as an adaptation to escape fire (Kaul *et al*., 2000), for example, *Amphicarpum amphicarpon* (as *Amphicarpon purshii*) (Cheplick & Quinn, 1987), *Commelina virginica* (Kaul *et al*., 2000) and *Vigna minima* (Gopinathan & Babu, 1987). In *Vigna minima*, a colonising species from the Western Ghats in India, Gopinathan & Babu (1987) argued that amphicarpy is primarily an adaptation to forest fires. *Amphicarpum amphicarpon* is an annual grass that grows in fire‐prone habitats in disturbed sandy areas on the Coastal Plain of the eastern USA from New Jersey to Georgia. Populations of this species respond positively to fire due to protection of subterranean seeds from high temperatures and post‐fire germination in an environment relatively free of competing perennial vegetation in the spring following experimental burns the previous autumn (Cheplick & Quinn, 1988*b*).

Amphicarpy also has been viewed as a possible adaptation to avoid seed predators and herbivores (Plitmann, 1973; Ellner & Shmida, 1981; Cheplick, 1987), i.e. the buried fruits/seeds would be protected. For example, *Lathyrus ciliolatus*, *Trifolium polymorphum*, *Vicia angustifolia* and *V. sativa* var. *platysperma* are distributed in heavily grazed sheep production areas (Kumar *et al*., 2012). Therefore, some amphicarpic species occur in habitats under biotic stress (overgrazing), while others are subject to physically stressful conditions (aridity, fire).

## V. CLEISTOGAMY AND BREEDING SYSTEM

In the amphicarpic *sensu stricto* species *Vigna minima* (Gopinathan & Babu, 1987), *Amphicarpaea bracteata* (Schnee & Waller, 1986; Trapp, 1988) and *A. edgeworthii* (Zhang *et al*., 2015), plants produce subterranean CL flowers and both aerial CL and CH flowers, whereas in the amphicarpic *sensu stricto* species *Amphicarpum amphicarpon* (McNamara & Quinn, 1977), *Cardamine chenopodifolia* (Cheplick, 1983) and *Commelina benghalensis* (Maheshwari & Maheshwari, 1955) plants produce CL flowers underground and CH flowers aboveground. On the other hand, amphicarpic *sensu lato* plants of *Catananche lutea* (Ruiz de Clavijo, 1995), *Gymnarrhena micrantha* (Koller & Roth, 1964; Plitmann, 1973) and *Emex spinosa* (Plitmann, 1973; Evenari *et al*., 1977) produce potentially outcrossing aerial CH flowers and fruits aboveground and potentially outcrossing aerial (near soil surface) CH flowers that are pulled into the soil shortly after they are insect‐pollinated and thus produce subterranean seeds (see Baskin & Baskin, 2017). In general, aerial CH flowers are large and brightly coloured and subterranean flowers small, white and invariably much reduced, with a small, non‐pigmented corolla enclosed in much‐reduced, scale‐like sepals (Speroni & Izaguirre, 2001, 2003; Zhang, Yang, & Rao, 2006; Kumar *et al*., 2012).

Aerial CH flowers are potentially outcrossed, while subterranean CL flowers are obligately self‐pollinated (Gopinathan & Babu, 1986; Schnee & Waller, 1986; Ruiz de Clavijo, 1995; Zhang *et al*., 2006; Kumar *et al*., 2012). However, CH flowers can also produce inbred seeds by selfing or by pollen from one CH flower fertilizing the ovules of another CH flower on the same plant (geitonogamous selfing) (Cheplick & Quinn, 1986; Trapp & Hendrix, 1988; Stewart, 1994; Zhang, Yang, & Rao, 2005; Liang *et al*., 2009; Speroni *et al*., 2009). Four breeding systems occur in the amphicarpic *sensu stricto* species *Vigna minima*: (*i*) cross‐pollinated CH flowers; (*ii*) cross‐ or self‐pollinated aerial pseudocleistogamy (i.e. closed and open flowers are morphologically similar, but those that do not open are self‐pollinated); (*iii*) self‐pollinated obligate subterranean true CL; and (*iv*) self‐pollinated subterranean pseudocleistogamy. In the latter breeding system, aerial shoots with flowers that do not open grow into the soil, thus placing the developing fruit underground (Gopinathan & Babu, 1987).

*Trifolium polymorphum* reproduces asexually by stolons and sexually by aerial CH and subterranean CL flowers (Real *et al*., 2007; Speroni *et al*., 2009, 2014). Aerial flowers are papilionaceous, with morphological features that are common to entomophilous flowers (i.e. large, brightly‐coloured, scent‐emitting, and with nectar at the base of the ovary), while the corolla of subterranean flowers is reduced to three petals and an androecium with only three stamens, whose anthers touch the stigma (Speroni & Izaguirre, 2001, 2003; Speroni *et al*., 2014). Both flower types have bisporangiate anthers, secretory cells, and a developed endothecium (Speroni & Izaguirre, 2001).

Ontogenetic studies have shown that the embryo sac does not develop apomictically in aerial and subterranean flowers of *T. polymorphum* (Speroni & Izaguirre, 2001; Speroni, Izaguirre, & Bernardello, 2010). Anthers of subterranean flowers never open, but the pollen tubes grow through the anther wall. Thus, subterranean seeds are produced *via* obligate self‐pollination. Anatomical studies of embryo sac ontogeny and egg cell development of aerial flowers revealed that zygote formation occurs before anthesis (Speroni & Izaguirre, 2001); therefore, self‐pollination also occurs in aerial CH flowers. However, studies of intrafloral phenology of *T. polymorphum* show that aerial CH flowers exhibit morphological and functional characteristics that promote outcrossing and delay selfing (Speroni *et al*., 2009). When the anthers dehisce prior to anthesis, the stigma is positioned above the anthers. Moreover, pollen viability of aerial CH flowers was maximal 1–2 days following anthesis, whereas stigmatic receptivity was maximal 3–4 days after anthesis. The authors concluded that *T. polymorphum* is an allogamous, self‐compatible species and that seed production is increased by pollinator visitation (Speroni *et al*., 2009, 2014).

In the non‐amphicarpic cleistogamous, mixed‐mating species *Impatiens pallida*, average effective selfing in CH flowers was >50% (Stewart, 1994). In the amphicarpic annual herb *Amphicarpaea bracteata*, the typical outcrossing rate was only about 0.5% or less (Parker, 1988). In the amphicarpic perennial herbs *Polygala lewtonii* (Swift *et al*., 2016) and *Trifolium polymorphum* (Real *et al*., 2007), self‐fertilization ranged from 80 to 93% and 60%, respectively. Mixed‐mating in amphicarpic species provides a fitness advantage through production of genetically diverse progeny *via* CH flowers, while preserving locally adapted alleles *via* CL flowers (Koontz *et al*., 2017). More biomass (energy) is required to produce CH than CL flowers, and CL flowers may produce more seeds than CH flowers (Schoen & Lloyd, 1984; Oakley, Moriuchi, & Winn, 2007; Winn & Moriuchi, 2009; Koontz *et al*., 2017). However, CL flowers increase the susceptibility of a population to genetic drift and inbreeding depression if deleterious alleles cannot be purged (Zeide, 1978; Oakley *et al*., 2007; see papers on purging by Crnokrak & Barrett, 2002 and Dart & Eckert, 2013). Fitness trade‐offs between CH and CL flowers help maintain amphicarpy as a mixed‐mating strategy (see Baskin & Baskin, 2017 and references cited therein). Furthermore, the genetic variation generated by CH outcrossing could facilitate adaptation of cleistogamous species to environmental change (Oakley *et al*., 2007).

## VI. LIFE‐HISTORY DIFFERENCES BETWEEN AERIAL AND SUBTERRANEAN SEEDS AND OF PLANTS DERIVED FROM THEM

### (1) Seed morphology and size

Generally, amphicarpic plants produce a large number of relatively small aerial seeds and a small number of relatively large subterranean seeds (Walker & Evenson, 1985*a*,*b*; Schnee & Waller, 1986; Cheplick, 1987; Kawano *et al*., 1990; Kaul *et al*., 2000; Speroni & Izaguirre, 2001; Zhang *et al*., 2015). Moreover, aerial and subterranean seeds differ in their morphology/structure. *Catananche lutea* (Asteraceae) produces five types of achenes: subterranean amphic‐1 and amphic‐2 and peripheral, intermediate and central in an aboveground capitulum. Both types of subterranean achenes are larger than the aerial ones and are not dispersed; amphic‐1 achenes do not have a pappus, whereas amphic‐2 achenes have a pappus (Ruiz de Clavijo, 1995).

Subterranean fruits of *Commelina benghalensis* are three‐seeded and aerial fruits five‐seeded (Maheshwari & Maheshwari, 1955). Kumar *et al*. (2012) compared aerial and subterranean seeds of three Australian endemic legumes, *Flemingia pauciflora*, *Glycine falcata* and *Vigna lanceolata*, and an exotic species, *Centrosema rotundifolium*. The aerial dehiscent pods of *F. pauciflora* were only two‐seeded, and those of the other three species were generally multi‐seeded and dehiscent. However, the subterranean pods of the four species were thin‐walled, non‐dehiscent and mostly one‐ or two‐seeded. The aerial seeds were smaller than the subterranean seeds, and in the three endemics they were generally dark‐coloured or speckled black. By contrast, the subterranean seeds were uniform in size and more lightly coloured (Kumar *et al*., 2012).

Aerial and subterranean seeds also may differ in seed coat anatomy. In *Amphicarpaea edgeworthii*, aerial seeds are dark brown and kidney‐shaped, while subterranean seeds are purple‐brown and either kidney‐shaped or irregular spherical. The aerial seed coat is composed of cuticle, a palisade cell layer (water‐impermeable), light line, hourglass cell layer and parenchyma cells and the seed coat of subterranean seeds of a ‘pre‐palisade’ cell layer (water‐permeable) and several layers of parenchyma cells. However, subterranean seeds did not have a cuticle or a light line (Zhang *et al*., 2015).

### (2) Seed dispersal

Amphicarpy involves more than one dispersal strategy in an individual plant (Table 1). Aerial seeds are generally telechorous, and they are dispersed away from the mother plant. However, burial of subterranean seeds near the mother plant is an effective way of ensuring atelechory, i.e. no dispersal (van der Pijl, 1982). The aerial fruits of *Gymnarrhena micrantha* are dispersed by wind (anemochory), while subterranean fruits never leave the site of the dead parent plant (Koller & Roth, 1964; Loria & Noy‐Meir, 1979/80). These authors stated that functionally subterranean fruits of *G. micrantha* could be considered as equivalent to dormant vegetative regenerative buds of a perennial.

Peripheral achenes of *Catanache lutea* have a weakly developed pappus that is subtended by the inner bracts of the capitulum, and they are dispersed when the capitula are released by the dead mother plant (short‐range dispersal). Central achenes have a highly developed pappus and are wind dispersed soon after maturity (long‐range dispersal). Some intermediate achenes are dispersed like the central ones, while others are dispersed like the peripheral ones. The two subterranean achenes (amphic‐1 and amphic‐2) are not dispersed (Ruiz de Clavijo, 1995; Ruiz de Clavijo & Jimenez, 1998). In *Amphicarpaea bracteata* (Trapp, 1988) and *A. edgeworthii* (Zhang *et al*., 2015), the dehiscent aerial fruits are dispersed ballistically >2 m, while those of the indehiscent subterranean fruits remain close to the mother plant. A similar phenomenon has also been observed in *Lathyrus setifolius* and *Pisum fulvum* from Israel (Mattatia, 1977*a*,*b*).

In general, it has been suggested that if there is a high cost to dispersal, such as a high likelihood of seeds moving to an unsuitable site (Bonte *et al*., 2012), a highly restricted dispersal system could evolve and lead to local adaptation (Cousens, Dytham, & Law, 2008). From the perspective of ecology and evolution, aerial seeds have relatively high dispersal ability, which facilitates their reaching new sites away from the mother plant, thus expanding the geographical area of the population and establishing new populations. On the other hand, subterranean seeds are formed/placed in the vicinity of parental microsites, thus maintaining populations in the safe environment (Koller & Roth, 1964; Ellner & Shmida, 1981; Cheplick, 1987, 1994). Furthermore, sibling competition could arise among seedlings that emerge from subterranean seeds because of the very restricted dispersibility of these seeds (Auld & Rubio de Casas, 2013; Hidalgo, Rubio de Casas, & Muñoz, 2016). The pronounced differences in dispersal of aerial and subterranean seeds may help ensure continued occupation of population sites in unpredictable environments in which seed production may not occur every year. In addition, high dispersal ability of the aerial seeds has the advantage of escaping from seed predators associated with the parent plant and of avoiding pathogens that may become established in dense stands of seedlings near the parent. In a mathematical model, Schoen & Lloyd (1984) showed how selection could favour dispersal dimorphism if CL seeds with limited dispersal are better able to establish within the maternal habitat and CH seeds that are widely dispersed are better able to escape deteriorating conditions of the local habitat; amphicarpic species were used to illustrate some of their arguments.

### (3) Seed germination

Aerial and subterranean seeds differ in degree (depth) of dormancy, dormancy‐breaking requirements and germination response to light/dark and temperature (Table 1). Subterranean and aerial achenes of the amphicarpic *sensu lato* desert annual *Gymnarrhena micrantha* germinated to higher percentages in light than in darkness (Koller & Roth, 1964). In subterranean seeds, the optimal temperature for germination was 15°C in both light and dark, while in aerial seeds 15°C was optimal only in light; germination percentages in darkness increased with a decrease in temperature (Koller & Roth, 1964).

Subterranean seeds of the amphicarpic *sensu lato* annual *Emex spinosa* (in Australia) were less dormant than aerial seeds, but scarification followed by treatment with gibberellic acid and kinetin overcame the physiological dormancy of both aerial and subterranean seeds. Germination of subterranean seeds of *E. spinosa* was less temperature dependent than that of aerial seeds. Aerial seeds required light to germinate to high percentages, but subterranean seeds germinated equally well in both light and dark (Weiss, 1980). However, in another study (in Israel) subterranean seeds of *E. spinosa* were more dormant than aerial seeds. Scarified (water‐permeable) subterranean seeds germinated to higher percentages in darkness than in light at constant temperatures from 5°C to 25°C, whereas scarified (water‐permeable) aerial seeds germinated equally well at all temperatures tested (10°C, 15°C, 20°C, 25°C, 30°C, and 35°C) in both light and darkness (Evenari *et al*., 1977).

In a recent review paper on seed germination of cleistogamous species, Baskin & Baskin (2017) reported the results of 65 case studies on the germination of aerial (A) *versus* subterranean (S) seeds for one amphicarpic *sensu stricto* species of Brassicaceae (*Cardamine chenopodiifolia*), one of Commelinaceae (*Commelina benghalensis*), four of Fabaceae (*Amphicarpaea bracteata*, *A. edgeworthii*, *Lathyrus ciliolatus* and *Vicia sativa* var. *amphicarpa*, all subfamily Papilionoideae) and one of Poaceae (*Amphicarpum amphicarpon*). For 49 (75.4%) of the case studies, A < S, for seven (10.8%) A = S and for nine (13.8%) A > S.

Interestingly, the desiccation‐tolerant aerial seeds of *Amphicarpaea bracteata* and *A. edgeworthii* have combinational dormancy (water impermeable seed coat plus a physiologically dormant embryo) that is broken by scarification followed by cold stratification, and the desiccation‐sensitive subterranean seeds have physiological dormancy that is broken by cold stratification (Zhang *et al*., 2015). These are the only cases we are aware of in which amphicarpic plants produce recalcitrant seeds.

### (4) Seed dispersal/dormancy strategy in (diaspore dimorphic) amphicarpic species does not fit the high‐risk/low‐risk strategy model

**Fig 4:**
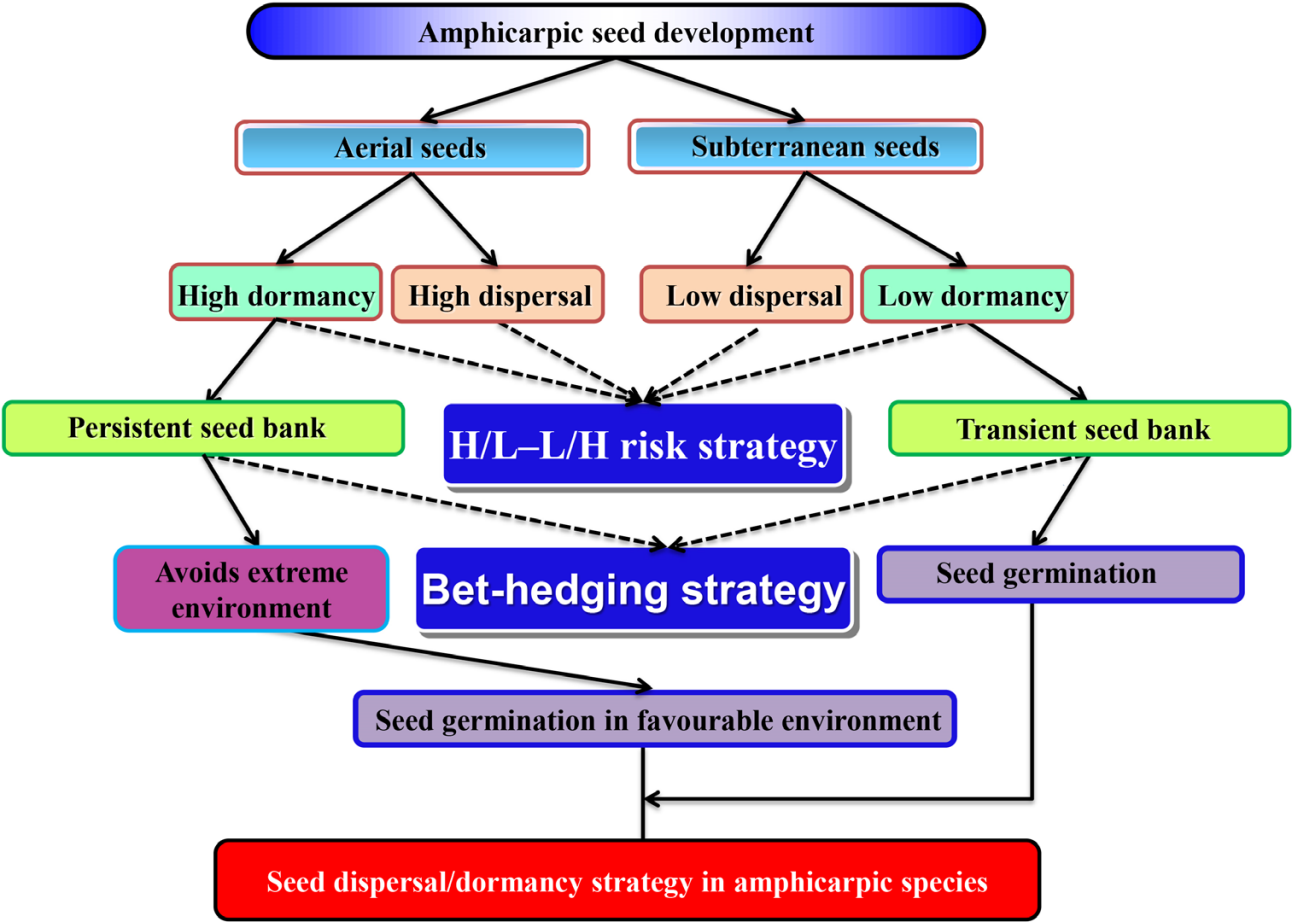
Seed dispersal/dormancy strategy in amphicarpic species. H/L, high‐risk dispersal/low‐risk dormancy; L/H, low‐risk dispersal/high‐risk dormancy.

In most non‐amphicarpic fruit/seed (diaspore) dimorphic species, there are two dispersal–dormancy strategies. One diaspore is easily dispersed and has low (or no) seed dormancy (i.e. high risk, H/H), while the second morph has one low (or no) dispersal ability and high (or relatively high) seed dormancy (i.e. low risk, L/L). Thus, the species has a high‐risk–low‐risk (H/H–L/L) strategy for seed dispersal/dormancy–germination (Venable, 1985; Baskin *et al*., 2013, 2014; Baskin & Baskin, 2014). Theoretically, the H/H morph can colonize new sites away from the mother plant shortly after dispersal, while the L/L morph remains near the mother plant and is slow to germinate due to time/conditions required for breaking dormancy and germination. However, in amphicarpic *sensu stricto* species *Amphicarpaea bracteata* (Schnee & Waller, 1986; Trapp & Hendrix, 1988), *A. edgeworthii* (Zhang *et al*., 2015) and *Amphicarpum amphicarpon* (as *Amphicarpon purshii*) (McNamara & Quinn, 1977) and in the amphicarpic *sensu lato* species *Emex spinosa* (Weiss, 1980) and *Gymnarrhena micrantha* (Koller & Roth, 1964), aerial seeds are dispersed for relatively longer distances (H, high risk) and have higher dormancy (L, low risk), whereas subterranean seeds have low dispersal ability (L) and relatively shallow seed dormancy (H). Thus, this species has a H/L–L/H risk strategy for diaspore dispersal and dormancy, which does not fit the high‐risk (H/H)–low‐risk (L/L) strategy (Zhang *et al*., 2015). A conceptual model of dispersal/dormancy strategy is summarized in Fig. 4, and we speculate that most amphicarpic species, at least those in the Fabaceae, fit this model.

### (5) Seedlings

Seed mass affects various aspects of plant life history, especially seedling ecology (Baskin & Baskin, 2014). Differences in seedlings from aerial and subterranean seeds often are associated with differences in seed mass. For a few amphicarpic species, a positive relationship between seed size and seedling survival has been found (Loria & Noy‐Meir, 1979/80; Cheplick & Quinn, 1987; Cheplick, 1994). Plants from subterranean seeds often have a competitive advantage (Weiss, 1980; Cheplick & Quinn, 1982) and are more resistant to stress (Koller & Roth, 1964; Evenari *et al*., 1977; Loria & Noy‐Meir, 1979/80; Weiss, 1980; Cheplick & Quinn, 1982; Cheplick, 1983; Trapp & Hendrix, 1988; Ruiz de Clavijo, 1995) than those from aerial seeds. Seedlings of *Gymnarrhena micrantha* (Koller & Roth, 1964; Evenari, Shanan, & Tadmore, 1971; Loria & Noy‐Meir, 1979/80) and *Cardamine chenopodifolia* (Cheplick, 1983) from subterranean seeds are much larger and more tolerant of low soil moisture and drought stress than those from aerial seeds; however, seedlings from subterranean seeds grow much slower than those from aerial seeds. Seedlings of *Polygonum thunbergii* (as *Persicaria thunbergii*) from subterranean seeds had 37% more mass and greater stem length, leaf number and root length than those from aerial seeds. Moreover, stem length of seedlings from subterranean seeds increased more rapidly than that of seedlings from aerial seeds, which allowed them to escape flooding conditions (Choo *et al*., 2015). By contrast, for another population of *P. thunbergii*, size of aerial and subterranean seeds was similar, and relative stem length growth rate, biomass allocation and biomass of seedlings from the two seed types did not differ between mother plants grown under different nutrient availability conditions (Kim, Nam, & Kim, 2016).

### (6) Development of fruiting structures

Although all amphicarpic species produce subterranean fruits/seeds, the way in which they are formed varies. The underground fruiting structures of the rhizomatous perennials *Flemingia pauciflora* and *Vigna lanceolata* develop on rhizomes that originate at underground cotyledonary nodes, whereas in *Glycine falcata* the rhizomes originate at the junction between the fleshy taproot and the stem. Rhizomes that emerged at the soil surface or from water‐drainage holes in the bottom of pots produced new ramets (Kumar *et al*., 2012). In *Cardamine chenopodifolia* (Cheplick, 1983, 1987; Fig. 1B), *Commelina benghalensis* (Walker & Evenson, 1985*a*,*b*), *C. forskalaei* (Maheshwari & Maheshwari, 1955) and *Polygala polygama* (Fig. 1C), all subterranean flowers are formed on subterranean stems. However, some of the subterranean flowers of *Amphicarpaea bracteata* (Darwin, 1888; Schnee & Waller, 1986) and *A. edgeworthii* (Zhang *et al*., 2005; Fig. 1A) are formed underground at the apices of short cotyledonary shoots and on aerial axillary shoots that arise from the first node and become buried in soil. By contrast, subterranean flowers/seeds of *Amphicarpum amphicarpon* (as *Amphicarpum purshii*) are formed on the tip of downward‐growing tillers. Each tiller ends in a much reduced inflorescence consisting of a single closed spikelet that matures only one seed (technically a caryopsis). The flowers of *Catananche lutea* (Ruiz de Clavijo, 1995; Fig. 1D), *Emex spinosa* (Evenari *et al*., 1977; Fig. 1E) and *Gymnarrhena micrantha* (Zamski, Ucko, & Koller, 1983) that give rise to subterranean fruits are formed aboveground, and contractile roots pull the basal part of the plant belowground after the basal fruits start to develop following insect pollination.

The *Vigna lanceolata* complex includes fibrous‐rooted annuals and tuberous‐rooted perennials as well as erect bushes and semi‐erect to prostrate vines and both aerial and amphicarpic reproduction. Amphicarpy has been observed in six morphotypes of this complex, among which two are fibrous‐rooted annuals and four tuberous‐rooted rhizomatous perennials (Lawn & Holland, 2003). In the four amphicarpic perennials, amphicarpy is habitual, and leafless underground stems or rhizomes give rise to fruiting structures. In the two annuals, specialized underground axillary geotropic stems originate along prostrate aerial stems, penetrate the soil and then produce seeds. One of the annuals exhibited habitual amphicarpy and the other opportunistic amphicarpy, i.e. underground fruiting structures developed only where aerial stems become covered with soil or leaf litter.

### (7) Post‐seedling growth/survival and seed production

Seed size also influences post‐seedling plant growth and reproductive output of amphicarpic species (Weiss, 1980; Cheplick & Quinn, 1982; Schnee & Waller, 1986). In general, plants from aerial and subterranean seeds differ in phenology, subterranean/aerial seed production ratio, seed mass, seed number and response to environmental change (Cheplick, 1987; Kawano *et al*., 1990; Sadeh *et al*., 2009; Choo *et al*., 2014; Zhang *et al*., 2017). Only relatively large plants growing under favourable conditions invest in aerial flowers and fruits, in which case plants growing in stressful conditions may produce only subterranean seeds. Plants of *Amphicarpaea bracteata* from the large subterranean seeds were much larger than those from the small aerial seeds, which made them more likely to produce aerial seeds (Trapp & Hendrix, 1988). Moreover, larger plants of *A. bracteata* are less subject to pathogen attack (Parker, 1986).

**Fig 5:**
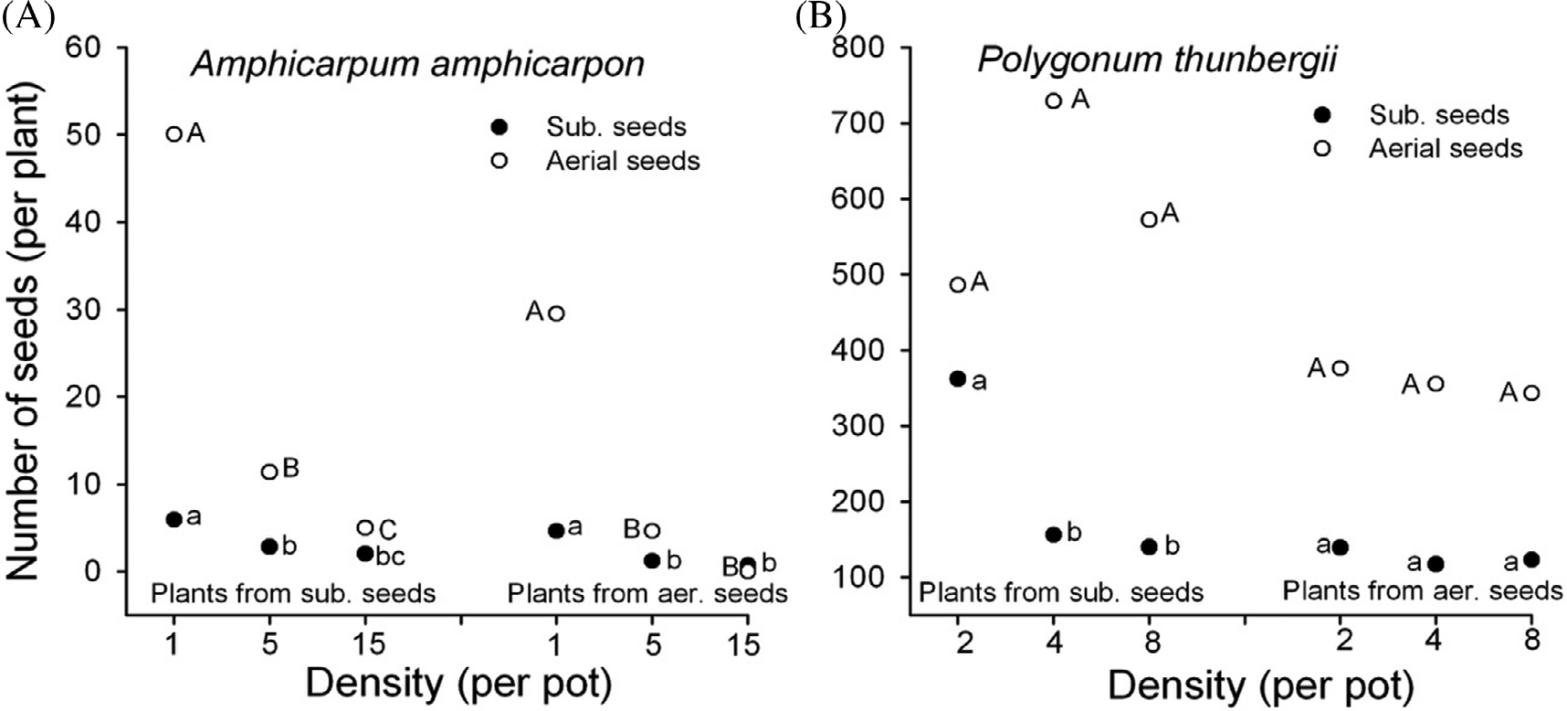
Mean number of subterranean and aerial seeds per plant in relation to density in two amphicarpic annuals. Plants of (A) *Amphicarpum amphicarpon* (Cheplick & Quinn, 1983) and (B) *Polygonum thunbergii* (Nam *et al*., 2017) were grown from subterranean or aerial seeds in monocultures. Density was per 11.4‐cm‐diameter pot in A and per 30‐cm‐diameter pot in B. Filled circles with different lowercase letters (subterranean seeds) and open circles with different uppercase letters (aerial seeds) indicate statistically significant differences (*P* < 0.05) among densities within a group.

Plants from aerial seeds of *Amphicarpum amphicarpon* (as *Amphicarpum purshii*) (McNamara & Quinn, 1977; Cheplick & Quinn, 1982) and *Cardamine chenopodifolia* (Cheplick, 1983) flowered later than those from subterranean seeds, while plants from subterranean seeds of *Commelina benghalensis* flowered later than those from aerial seeds (Walker & Evenson, 1985*a*). Plants from the relatively small aerial seeds of *A. amphicarpon* (as *Amphicarpum purshii*) were more sensitive to competition than those from the large subterranean seeds. At a density of 15 plants per pot (11.4 cm diameter), plants from aerial seeds did not produce aerial spikelets, whereas at densities of 5, 3 and 1 plant(s) per pot 8, 85 and 100% of the plants produced aerial spikelets (Cheplick & Quinn, 1983). When subterranean seeds were planted at a density of 30, 15, 5 or 1 per pot 24, 43, 90 and 100% of the resulting plants, respectively, produced aerial spikelets. Unpublished data from the Cheplick & Quinn (1983) study on numbers of the two seed types in relation to density are shown in Fig. 5A. Aerial seed production was greatly reduced compared to that of subterranean seeds as density was increased in monocultures of plants from both aerial and subterranean seeds. In *Polygonum thunbergii*, plants from subterranean seeds also produced more seeds of both types than plants from aerial seeds; however, seedling planting density did not significantly affect aerial seed production (Nam, Kim, & Kim, 2017; Fig. 5B).

The abiotic and biotic environment, geographic location and plant size can influence the ratio of aerial and subterranean seeds produced by amphicarpic plants. With a decrease in amount of light, water and nutrients, the number of aerial seeds decreased significantly more than that of subterranean seeds in several amphicarpic species (Weiss, 1980; Schnee & Waller, 1986; Cheplick, 1988, 1994; Cheplick & Quinn, 1988*b*; Nam *et al*., 2017; Zhang *et al*., 2017). With an increase in nutrient availability, total number of spikelets and seed mass of *Amphicarpum amphicarpon* (as *Amphicarpum purshii*) increased, but changes were greater for aerial than for subterranean reproduction. Biomass allocation to subterranean reproduction of this species was not affected by nutrient availability (Cheplick, 1989). Because *A. amphicarpon* (as *Amphicarpon purshii*) size also was reduced significantly with density, the allometry of CH (aerial)/CL (subterranean) ratio to vegetative dry mass tended to increase up to a point, but then levelled off to where biomass allocation to CH was about 60% of that allocated to CL in the largest plants (Cheplick, 2007). It was also noted that CH allocation was more variable than CL allocation at low, medium and especially high density, as quantified by coefficients of variation.

In species that produce both CH and CL, the ratio of CH/CL flowers can also be affected by biotic and abiotic factors such as light level, nutrient availability and herbivory (Fukui & Takahashi, 1975; Culley & Klooster, 2007). Production of CL flowers is often favoured under stressful growth conditions (Cheplick, 1987; Trapp & Hendrix, 1988), apparently because they are energetically less costly to produce than CH flowers (Culley & Klooster, 2007; see Baskin & Baskin, 2017). With an increase in density in *A. amphicarpon* (as *Amphicarpum purshii*), dry mass allocated to CL was remarkably constant, varying from only 13 to 16%. By contrast, CH allocation declined from 8% in the control to 0.6% at the highest density. Thus, the CH/CL ratio decreased precipitously with increasing density (Cheplick, 2007).

Changes in flower type during development are due to alterations in the initial primordial buds (Culley & Klooster, 2007). In *Amphicarpaea edgeworthii*, aerial CH flowers are papilionaceous, consisting of five sepals, five petals, 10 (9 + 1) stamens and a solitary carpel in the centre. Aerial CL and subterranean CL flowers are composed of sepals, five rudimentary petals, two stamens that produce anthers and a single carpel. Using scanning electron microscopy (SEM), Zhang *et al*. (2006) found little difference in organ initiation among the three kinds of flowers (i.e. aerial CH, aerial CL and subterranean CL). At the mid‐ to late‐development stage, development of the carpel and the petals and stamens in the aerial and subterranean flowers diverged, at which time aerial CH flowers become papilionaceous but CL flowers do not (Zhang *et al*., 2006).

In *Polygonum thunbergii*, aerial seed mass decreased along a gradient of decreasing light availability, but subterranean seeds were not affected (Kawano *et al*., 1990). Experimental shading also reduced number and mass of aerial but not of subterranean seeds in *P. thunbergii* (Nam *et al*., 2017). Production of subterranean seeds of *Emex spinosa* was less affected by density than that of aerial seeds. In mixtures of plants reared from aerial and subterranean seeds, plants from subterranean seeds had a larger leaf area and larger stems and produced more aerial seeds than plants from aerial seeds; these differences were not apparent in monocultures of plants from subterranean seeds (Weiss, 1980). However, with an increase in nitrogen availability, allocation to aerial seed production in *E. spinosa* increased from 7 to 46%, whereas allocation to subterranean seeds decreased from 38 to 3% (Weiss, 1980). Thus, production of both aerial and subterranean seeds in this species is quite plastic and exhibits considerable adjustment to abiotic conditions (Cheplick, 1994).

Under limited resources (abiotic stress), amphicarpic plants produce subterranean seeds first (Weiss, 1980; Cheplick & Quinn, 1982; Kawano *et al*., 1990), and the aerial/subterranean seed ratio is low. However, under favourable conditions the aerial/subterranean seed ratio is high (Koller & Roth, 1964; Cheplick & Quinn, 1983; Kaul, Sharma, & Koul, 2002; Sadeh *et al*., 2009). Cheplick (1994) concluded that production of aerial seeds is more plastic than that of subterranean seeds. The ability of amphicarpic species to shift the aerial:subterranean seed production ratio may increase fitness over generations by reducing variance in number of offspring left per generation (*R*_o_), a requirement for bet‐hedging (Simons, 2011).

### (8) Survival strategies

Early production of subterranean CL seeds provides reproductive assurance (i.e. obligate self‐pollination in CL flowers results in high seed set), whereas later production of aerial seeds allows plants to increase reproductive output near the end of the growing period according to the availability of resources (Cheplick, 1989). Because there is no guarantee of survival of the plant in an unpredictable environment, subterranean seeds are produced as soon as possible: a ‘pessimistic strategy’. On the other hand, if there is little or no risk to future survival of the plant, yield is maximized in the late vegetative stage by production of aerial seeds: an ‘optimistic strategy’ (Zeide, 1978). For example, formation of subterranean fruits in *Gymnarrhena micrantha* follows a pessimistic strategy, which ensures that some seeds mature; the number of fruits produced is independent of plant mass (Zeide, 1978). However, production of aerial fruits in *G. micrantha* follows an optimistic strategy, i.e. number of fruits increases with increase in plant mass. In *Polygonum thunbergii*, flooding changed biomass allocation to earlier aerial flowering, with potential impacts on the population (Choo *et al*., 2014). In this species, aerial flowering starts earlier than subterranean flowering (Kim *et al*., 2016) in contrast to other amphicarpic species. The authors suggested that there were different evolutionary drivers (e.g. flooding) for seed allocation in *P. thunbergii*, a wetland species, than for amphicarpic species of arid habitats.

### (9) Soil seed bank

A persistent soil seed bank may be crucial for adaptation of plant species to unpredictable environments, and it plays an important role in persistence of populations and contributes to future genetic variability (Baskin & Baskin, 2014). Cheplick & Quinn (1988*b*) showed that fire killed aerial seeds, but not subterranean seeds, of the amphicarpic species *Amphicarpum amphicarpon* (as *Amphicarpum purshii*). Thus, after fire all emerging seedlings were from subterranean seeds (Cheplick & Quinn, 1988*b*). In *Amphicarpaea bracteata* and *A. edgeworthii*, subterranean seeds form a transient seed bank and aerial seeds a persistent seed bank (Zhang *et al*., 2015). In most amphicarpic species, the seed bank has not been well described, but the limited available evidence suggests that aerial seeds have greater dormancy and perhaps contribute to a persistent seed bank, while subterranean seeds have low dormancy and contribute to the transient seed bank (Cheplick, 1994).

## VII. GENETICS AND QUANTITATIVE GENETIC VARIATION

Plants with breeding systems that are a mixture of selfing and outcrossing have reduced levels of genetic diversity within and greater levels among populations (Hamrick & Godt, 1997). Using isozyme electrophoresis, Marshall & Weiss (1982) demonstrated that Australian *Emex spinosa* is genetically homogeneous within populations and genetically different among populations. The genetic uniformity of local populations of *E. spinosa* suggests that this species predominantly is self‐pollinated. Analysis of 11 microsatellite loci in the amphicarpic species *Polygala lewtonii* also revealed high levels of inbreeding (Swift *et al*., 2016). Using simple sequence repeats (SSRs), Zhang *et al*. (2005) demonstrated that selfing is more common than outcrossing in populations of *A. edgeworthii*. Liang, Yang, & Rao (2007) used random amplified polymorphic DNA (RAPD) to evaluate the level and pattern of genetic variation in 15 populations of *A. edgeworthii*. The results revealed a high level of genetic differentiation among, but not within, populations. Thus, the pattern of genetic structure in *A. edgeworthii* matches that of an inbreeding species.

Populations of *A. bracteata* vary substantially in disease resistance, enzyme variants and leaf morphology throughout its range. Such differences are an expected consequence of the restricted recombination associated with the mating system of *A. bracteata* (Parker, 1991). An isozyme analysis of 978 plants from 33 populations of *A. bracteata* in seven states of the USA (greatest distance between sites was 1000 km) revealed that three cryptic taxa designated as lineages I_a_, I_b_, and II could be distinguished. At seven of 18 electrophoretic loci, lineages I and II did not have any alleles in common, while lineages I_a_ and I_b_ differed at one locus (Parker, 1996). When the two dominant lineages are mixed by outcrossing, the progeny have inferior performance relative to both parental types. The reproductive success of F_3_ hybrid plants used in a common garden study was about half that of the parental biotype average, which implies that severe outbreeding depression outweighed any positive contribution of heterosis to the fitness of hybrid plants (Parker, 1991).

Kartzinel *et al*. (2016) characterized single nucleotide polymorphism (SNP) variation in 128 individuals of *A. bracteata* from southern Wisconsin (USA) and assessed the within‐ and among‐population variation at 3928 SNPs. They found three strongly divergent and highly inbred genetic groups showing little relation to site location. However, estimates of among‐group migration were low, and <2% of the individuals were hybrids. In fact, the U‐shaped distribution of pairwise among‐population genetic differentiation coefficient (*F*_ST_) values seemed to indicate centres (‘islands’) of genomic divergence. These islands may be associated with hybrid incompatibility loci that arose *via* allopatry (Kartzinel *et al*., 2016).

Although selfing is an important component of the breeding system of many amphicarpic species, considerable phenotypic variability in quantitative traits exists in populations in which occasional outcrossing occurs and/or individuals are phenotypically plastic (Cheplick & Quinn, 1986, 1988*a*). In 10 maternal families (i.e. progeny derived from the same maternal parent) of *Amphicarpum amphicarpon* (as *Amphicarpum purshii*), 60% of the phenotypic variation was found within families for all characters examined in plants from seeds of either bagged (self‐pollination) or open (potentially cross‐pollination) panicles (Cheplick & Quinn, 1986). Thus, the mixed CH/CL breeding system of amphicarpic species that allows for some outcrossing coupled with a highly plastic phenotypic response to environmental variables explains the maintenance of considerable genotypic and phenotypic variation in these species. Some theoretical considerations for maintenance of a mixed CH/CL breeding system in cleistogamous species can be found in Baskin & Baskin (2017).

Subterranean and aerial CL flowers offer reproductive assurance when pollinators are rare or absent. Consistent selfing can eliminate (purge) deleterious recessive alleles within populations over time, which could lead to a decrease in the level of inbreeding depression (Oakley *et al*., 2007). In *Amphicarpum amphicarpon* (as *Amphicarpon purshii*), selfing is a major component of the breeding system due to the subterranean CL flowers and to the potential for selfing in wind‐pollinated aerial CH flowers. Seed viability, germination and fitness of progeny did not differ between seeds produced on open *versus* bagged aerial panicles (Cheplick & Quinn, 1983). Similar results were found by Speroni *et al*. (2014) from a total of 210 hand‐pollinated aerial flowers of the amphicarpic *sensu stricto* species *Trifolium polymorphum*. Pollen tube growth rate and seed production of self‐pollinated CH flowers were higher than they were for cross‐pollinated CH flowers, but the seeds produced after self‐ and cross‐pollination did not differ in size or germination. Subterranean flowers may increase seed production in the event that aerial flowers are not pollinated, while recombination of new alleles in outcrossed aerial progeny may cause them to exhibit heterosis (Culley & Klooster, 2007).

Some components of the amphicarpic reproductive strategy show significant quantitative genetic variation among families (Cheplick & Quinn, 1986), but these are not the traits most closely allied to fitness (Cheplick & Quinn, 1988*a*). Low variation in fitness‐related traits could reflect that genetic fixation and/or developmental canalization has occurred for some traits such as those related to subterranean seed production. Among 60 quantitative characters of 12 maternal families of *Amphicarpum amphicarpon* (as *Amphicarpum purshii*) reared from aerial seeds, only 19 characters differed significantly in between‐family phenotypic variation. Total variation due to (maternal) family was 14.9%. The highest narrow‐sense heritabilities were for biomass allocation and vegetative characters, while subterranean seed set and mass of both seed types had the lowest genetic variation (Cheplick & Quinn, 1988*a*). Since number and mass of CL seeds correlate with shoot mass across maternal families in *A. amphicarpon* (as *Amphicarpum purshii*) (Cheplick, 1994), directional selection on shoot mass may indirectly select for increased subterranean (but not aerial) seed output.

## VIII. ECOLOGICAL AND EVOLUTIONARY CONSEQUENCES OF AMPHICARPY

**Fig 6:**
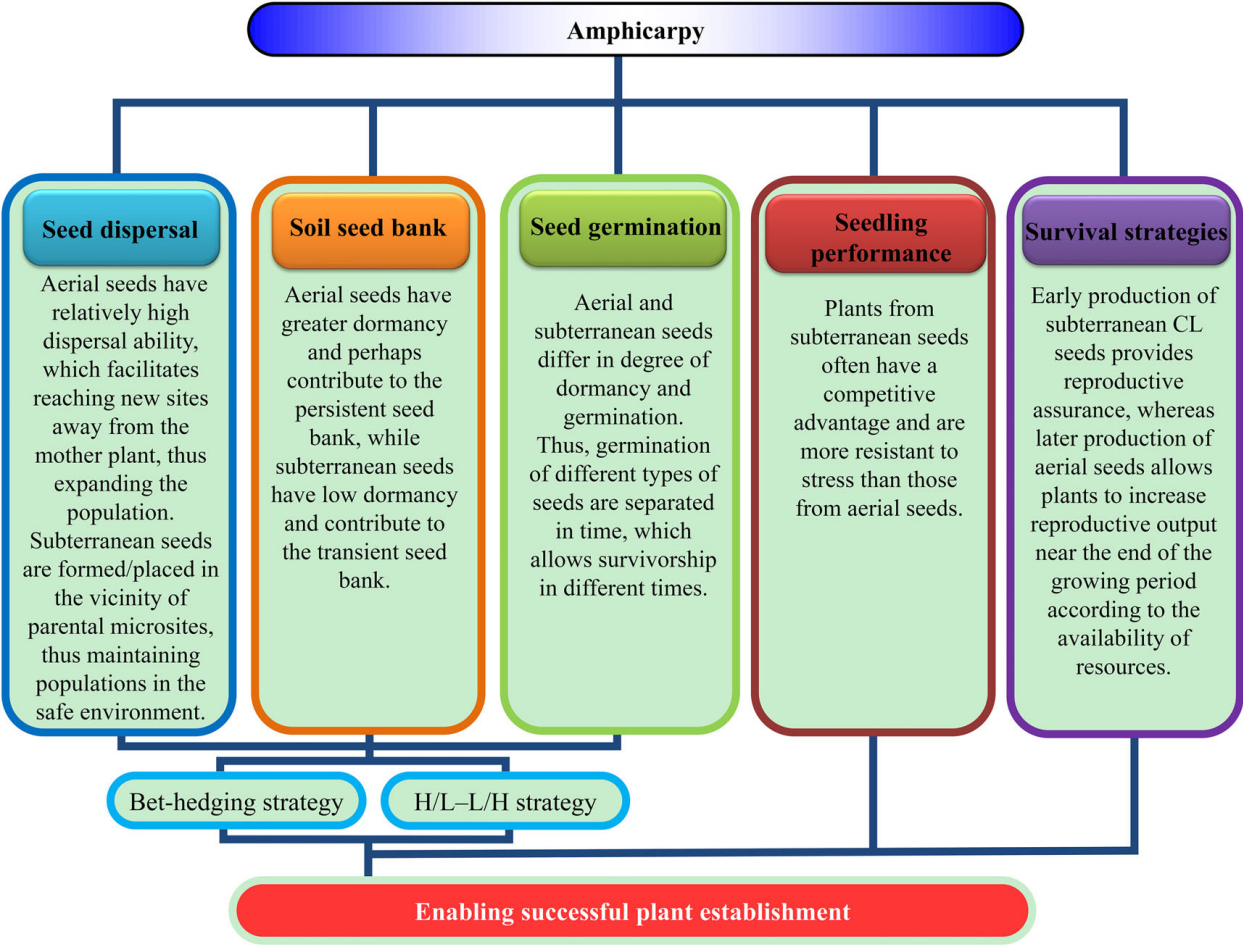
Ecological advantages of amphicarpic plants. CL, cleistogamous; H/L, high‐risk dispersal/low‐risk dormancy; L/H, low‐risk dispersal/high‐risk dormancy.

For amphicarpy to evolve and be maintained, subterranean fruits must exhibit fitness advantages distinct from those of aerial fruits. That is to say, for the two morphs to evolve and be maintained in the population or species, the fitness advantage to a plant producing both morphs must be greater than that of it producing only the aerial morph or only the subterranean morph (Lloyd, 1984; Venable, 1985). Various suggestions that have been made from an ecological perspective to help explain the ecological (thus fitness) advantages of plants that produce subterranean fruits (Fig. 6), in addition to aerial fruits, are discussed below.

1. Burial of subterranean seeds affords partial protection from extremes of heat/cold and predators at the soil surface. Compared with aerial seeds, which often remain exposed on the soil surface, subterranean seeds can remain viable in relatively moist soil near the mother plant in arid environments, which protects the germinated seedlings from rapid dehydration and thus is beneficial to their successful establishment (Cheplick, 1987, 1994). In *Amphicarpum amphicarpon* (as *Amphicarpum purshii*), few aerial seeds produce seedlings successfully because the seedlings on the soil surface often lose water, leading to death. However, subterranean seeds, which are produced 3.5 cm belowground, may remain viable and established seedlings to a high percentage (Cheplick & Quinn, 1987; Kaul *et al*., 2000). In addition, seeds buried in subterranean microsites may escape death from high temperatures generated on the soil surface by fast‐moving fires (Cheplick & Quinn, 1988*b*; Kaul *et al*., 2000). At the same time, fire changes the original habitat and growth conditions, creating a suitable environment for seed germination and seedling growth for some species.
2. Subterranean fruits buried in the vicinity of the mother plant allow for a reduced investment in the energy cost of dispersal (Ellner & Shmida, 1981; Kaul *et al*., 2000; Bonte *et al*., 2012). Regardless of the habitat, subterranean seeds remain near the mother plant, where conditions are favourable for seed germination and seedling recruitment (Gopinathan & Babu, 1987; Trapp, 1988), and thus presence of the species can be maintained in the maternal habitat. On the other hand, aerial seeds often are dispersed away from the maternal plant and may reach additional habitats suitable for seedling establishment (Cheplick, 1994).
3. Referring to amphicarpic *sensu stricto* species, CL flowers are energetically less costly to produce in terms of biomass than CH flowers (Oakley *et al*., 2007; Winn & Moriuchi, 2009; see Baskin & Baskin, 2017), resulting in more resources available for seed production. Further, in addition to providing reproductive assurance, the obligately selfing subterranean (CL) flowers have a selfing advantage over CH flowers. In CL flowers both sets of genes (pollen and ovules) are passed on to the progeny, whereas in outcrossing CH flowers only one set of genes (ovule) is passed on to offspring (Culley & Klooster, 2007). At the individual plant level, then, amphicarpic *sensu lato* species have a 3:2 (50%) gene transmission advantage over a purely outcrossing plant with CH flowers. Thus, an amphicarpic *sensu lato* plant contributes two sets of genes to its own offspring and one set, *via* pollen, to the offspring of others, while an outcrossing CH plant contributes one set of genes to its own progeny and one to the progeny of others.
4. Amphicarpic plants exhibit a high degree of plasticity during aerial and subterranean reproduction. Subterranean fruits usually are formed earlier than aerial ones, and plants can change from production of aerial to subterranean propagules based on the current environmental conditions (Zeide, 1978; Cheplick, 1994; Sadeh *et al*., 2009).

Disadvantages of subterranean CL seed production include a decrease in genetic variation and genetic drift if the population is small, potentially high levels of inbreeding depression and an increase in sibling competition among seedlings from subterranean seeds that remain close to the mother plant (Cheplick, 1994). Disadvantages of CH aerial flower production include a high energetic cost in some species in which showy petals and copious pollen and nectar are produced, reliance on pollinators for pollination/fertilization and a possible cost of dispersal, e.g. moving to sites unsuitable for germination and establishment, or the mother plant spending energy on expensive dispersal features like high levels of sugars in a fruit to attract animal dispersers (Bonte *et al*., 2012).

## IX. EVOLUTION OF AMPHICARPY

Subterranean seed production has evolved independently in phylogenetically widespread taxa, and thus the relevant selection pressure is likely to differ from species to species (Cheplick, 1987; Kaul *et al*., 2000). Severe stress from herbivores and the physical environment (fire, drought) may be strong evolutionary forces that promote the evolution of amphicarpy in various habitats (Ellner & Shmida, 1981; Cheplick, 1987). Cheplick (1994) hypothesized that *Amphicarpum amphicarpon* (as *Amphicarpum purshii*) originated from a subtropical rhizomatous perennial clonal herb such as *A. muhlenbergianum*. As the perennial grass species migrated northward, it encountered a shorter growing season and restricted resources, and there was a reduction in the number of ramets and seeds produced. Consequently, *A. amphicarpon* evolved into an annual with short, subterranean tillers. With further reduction in ramet growth in response to a shorter growing season and adverse environmental conditions such as fire and dry soil, *A. amphicarpon* eventually evolved into an amphicarpic annual. This occurred because fire (or another disturbance) or desiccating conditions destroyed seeds produced above the soil surface. Thus any genotypes with a tendency to produce seed‐bearing tillers belowground had a selective advantage in the fire‐prone habitats where this species occurs (Cheplick, 1994).

From a perspective of evolution, if one phenotype (e.g. aerial diaspore) has the highest fitness in alternative environments, then constant expression of this phenotype will be favoured over alternative phenotypes (subterranean diaspore) (West‐Eberhard, 2003). However, under this scenario, if environmental extremes exceed the tolerances of the phenotype, then it will inevitably go extinct (Moran, 1992; West‐Eberhard, 2003; Auld & Rubio de Casas, 2013; Hidalgo *et al*., 2016). Thus, subterranean seed production in amphicarpic species may be symbolized as an adaptive trait with alternative fitness peaks.

Various mathematical models and empirical data have suggested that natural selection might often favour a dispersal dimorphism and mixed mating system under highly unpredictable environments. Using mathematical and computational modelling, Hidalgo *et al*. (2016) showed that high dispersal ability is adaptive when unpredictability of the environment is low and inbreeding depression high and that a dimorphic dispersal system and a mixed mating system, i.e. amphicarpy, are adaptive under high environmental unpredictability, especially if inbreeding depression is low. Furthermore, they found that populations with a single dispersal and mating system inevitably go extinct under high environmental stress and high inbreeding depression, whereas populations with mixed strategies were maintained under extreme environmental conditions (Hidalgo *et al*., 2016).

In amphicarpic species, aerial and subterranean seed morphs differ in dispersal and mating systems, and they have high fitness at some time or place. Thus, production of aerial and subterranean seeds represents the optimal strategy in a wide variety of contexts by ensuring that at least some offspring can function appropriately in a variety of environmental conditions (Venable, 1985; Auld & Rubio de Casas, 2013; Hidalgo *et al*., 2016). Amphicarpy decreases variance in the number of offspring produced per year and thus increases the geometric mean of the number of offspring across generations, i.e. by bet‐hedging (Venable, 1985; Simons, 2011).

## X. USE OF AMPHICARPIC LEGUMES IN AGRICULTURE

Worldwide, 90% of human caloric needs are supplied by only 20 species, with 60% of the global crop output coming from wheat (*Triticum aestivum*), maize (*Zea mays*), rice (*Oryza sativa)* and soya (*Glycine max*) (Massawe, Mayes, & Cheng, 2016). The integration of nutrient‐rich new or orphan (understudied/under‐utilized) crops into food systems is recognized to have an important role in agriculture (Tadele, 2014, 2019). Legumes can fix atmospheric nitrogen and convert it to ammonium (Varshney *et al*., 2012; Tadele, 2019), and many amphicarpic legumes have excellent nutritional profiles. For example, the chemical composition of both aerial and subterranean seeds of *Amphicarpaea bracteata* (peña *et al*., 1999) and *a. edgeworthii* (Jiang, Xu, & Ma, 2006) is similar to that reported for other food legumes. Moreover, the seeds of these two species have a higher protein content than either Bambara groundnut (*Vigna subterranea*) or groundbean (*Macrotyloma geocarpum*) (Duke, 1981). In comparison with FAO guidelines, seeds of *A. breacteata* are deficient in sulfur amino acids but not in tryptophan, which is higher than the FAO requirement (peña *et al*., 1999). In *Vigna minima*, levels of essential amino acids are markedly higher than the FAO/WHO standard, except for sulfur amino acids (Gopinathan *et al*., 1987). Therefore, amphicarpic legumes could help to protect world food supplies, particularly as challenges such as increasing world population, and climate change, diseases and pests threaten global food crop monocultures (Tadele, 2014).

In the tropics, legumes have a high potential for use as forage plants to improve pastures, prevent soil erosion and improve soil fertility (Cocks, 1999). However, the potential impact of tropical pasture legumes on livestock production is limited by lack of persistence when grown together with grasses and subjected to grazing (Cocks, 1999). Thus, maintenance of a soil seed bank plays a major role in their use (Lawn *et al*., 2016). It has been suggested that the production of subterranean seeds has the potential to keep the grazing‐independent soil seed bank replenished (Schultze‐Kraft, Schmidt, & Hohn, 1997). For example, production of subterranean seeds is a key trait in the ability of the geocarpic species *Trifolium subterraneum* to persist in sheep‐production areas of southern Australia. However, subterranean seeds are difficult to harvest, as in *T. subterraneum*, which limits the wide use of this species (Morley, 1961). The potential value of amphicarpy in a species includes the benefits of geocarpy and the production of aerial seeds that can be easily harvested.

Amphicarpy has been reported to be agriculturally important in the tropical forage legume genera *Centrosema*, *Flemingia*, *Glycine*, *Lathyrus*, *Macroptilium*, *Pisum*, *Trifolium*, *Vicia* and *Vigna*, which also contain important tropical pulse crops (Schultze‐Kraft *et al*., 1997; Cocks, 1999; Speroni & Izaguirre, 2003; Kumar *et al*., 2012; Lawn *et al*., 2016). In eastern Venezuela, a destructive harvest of six *Centrosema rotundifolium* accessions yielded 920–1900 kg of subterranean seeds/ha; seed yields from aerial pods were 13–856 kg/ha (Schultze‐Kraft *et al*., 1997).

The main agronomic value of amphicarpy is its role in promoting establishment and persistence of forage species in stressful environments (Cocks, 1999). In heavily grazed, drought‐prone regions of temperate west Asia and north Africa, production of subterranean seeds by *Vicia sativa* ssp. *amphicarpa* make the species less vulnerable to being grazed out, while the aerial seeds allow it to spread to new areas (Cocks, 1999; Lawn *et al*., 2016). The tuberous roots of *Macroptilium panduratum* have meristems from which new growth can occur after the aboveground biomass has been destroyed (Schultze‐Kraft *et al*., 1997).

*Trifolium polymorphum* is a southern South America stoloniferous, amphicarpic legume (Speroni & Izaguirre, 2003; Speroni *et al*., 2009, 2014). The species is an important component of natural pastures during winter because it produces good quality fodder, particularly for sheep (Speroni & Izaguirre, 2003). In addition to the soil seed bank formed by subterranean seeds, the meristems on the stolons are below the level of grazing by herbivores, which promotes persistence during the time of vegetative development. Therefore, although the species can be heavily grazed, production of subterranean seeds and accumulation of reserves in the stolons allows it to survive even in soils with a high risk of drought and with continuous, intensive and selective grazing by sheep (Coll & Zarza, 1992; Speroni & Izaguirre, 2003). Moreover, amphicarpy enables the plants to adapt to stressful environmental conditions by changing the allocation of resources from underground to aboveground reproduction and *vice versa* (Section VI.(7)). Thus, as with other traits that facilitate plant persistence (e.g. water‐impermeable seeds) amphicarpy might conceivably be an undesirable trait in systems where persistence is not wanted.

Amphicarpy also may be a useful trait for ensuring the persistence of legumes used as cover crops in association with tree crops or plantation crops. In the Australian sugar industry, the geocarpic species *Arachis pintoi* is a useful companion crop under trees and in sugarcane fallows, because its seedlings can emerge through the layer of litter left on the soil surface after sugarcane is harvested (Garside, Bell, & Magarey, 2001). Byth, Clements, & Syme (1980) suggested that the introduction of genes for amphicarpy from *Macroptilium panduratum* into the tropical pasture legume *M. atropurpureum* might be an innovative way to improve persistence of the latter species in tropical pastures.

If species with amphicarpy are shown not to be suitable for direct use, they may serve as donors of genes for plant breeding programs in other economically important closely related species. Of the Australian amphicarpic legumes, the endemic *Vigna lanceolata* is the most diverse (Redden *et al*., 2015) and includes fibrous‐rooted annuals, tuberous‐rooted perennials, erect bushes and semi‐erect to prostrate vines, and it has both aerial (only) and amphicarpic reproduction (Lawn & Bielig, 2016). Of the 56 parental combinations in a diallelic cross with eight accessions of seven perennial morphotypes, amphicarpy was routinely expressed in the hybrids, indicating that development of perennial accessions with potential for use as forages should be relatively straightforward (Lawn *et al*., 2016). Many of the F_1_ hybrids were self‐sterile. However, hybrids that were at least partially fertile produced many more subterranean than aerial seeds. Thus, amphicarpy plays an important role in perpetuating hybrids that might otherwise not set seeds and thus not survive. F_1_ hybrids between the two annual morphotypes exhibited habitual amphicarpy, indicating that it should be relatively straightforward to transfer genes for habitual amphicarpy into the more widely adapted and generally more vegetatively vigorous morphotypes (Lawn & Bielig, 2016).

The genus *Glycine*, to which the economically important crop soybean (*G. max*) belongs, includes two species with ‘habitual’ amphicarpy (*G. falcata* and *G. albicans*) and five with ‘opportunistic’ amphicarpy (*G. arenaria*, *G. hirticaulis*, *G. pindanica*, *G. pullenii* and *G. tomentella*) (Tindale & Craven, 1988, 1993; Pfeil & Craven, 2002; Kumar *et al*., 2012). These species differ greatly in growth form and ability to tolerate stress (Hymowitz & Newell, 1981; Pfeil & Craven, 2002), and thus they occur in a wide variety of habitats in Australia, the western Pacific and China (Hymowitz & Newell, 1981; Brown *et al*., 1985). In addition, they have various agriculturally favourable characteristics that do not occur in *G. max* (Hymowitz & Newell, 1981; Riggs *et al*., 1998; Graham & Vance, 2003), including resistance to various diseases (Graham & Vance, 2003), drought and salt tolerance (Graham & Vance, 2003) and tolerance to several herbicides (Brown *et al*., 1985; Singh & Hymowitz, 1999; Graham & Vance, 2003).

Transfer of these characters into the gene pool of cultivated soybean would greatly enhance the diversity of the soybean germplasm. However, the genetic diversity of amphicarpic *Glycine* species has not been fully explored by soybean breeders. Newell & Hymowitz (1983) performed hybridization studies within and between wild perennial *Glycine* species and found that pod set was 11% and 8% for intraspecific and interspecific crosses, respectively. The derived fertile diploid hybrid from soybean × *G. tomentella* (2n = 78) was resistant to the soybean cyst nematode *Heterodera glycines* (Riggs *et al*., 1998). A colchicine‐doubled F_1_ hybrid (2n = 118) between soybean (2n = 40) and *G. tomentella* (2n = 78) produced approximately 100 F_2_ seeds, and most F_2_ plants had a high level of fertility, although two of 24 plants had low pollen viability and a large number of fleshy pods (Shoemaker *et al*., 1990). These results suggest that transfer of economically beneficial genes into soybean from *G. tomentella* is possible.

## XI. IMPLICATIONS FOR FUTURE RESEARCH

Amphicarpy is a special kind of reproductive strategy, and a better understanding of it will contribute to knowledge of the ecology and evolution of plant life history and plant sexual reproduction, given that amphicarpic species share several life‐history attributes with non‐amphicarpic flower‐ and diaspore‐dimorphic species (Table 3). For example, studies of amphicarpic plants could shed light on the adaptive significance of mixed mating systems (Goodwillie *et al*., 2005) and dimorphic seed production (Imbert, 2002). For the most part, research on amphicarpy has focused mainly on comparison of the morphology of aerial and subterranean seeds, seed dormancy and germination, seed dispersal and biomass and reproduction allocation in mature plants derived from aerial and subterranean seeds. Several important aspects of the ecological functions of amphicarpy have been overlooked or given little attention, such as seed development and maturation and maintenance of a soil seed bank. Further research on these aspects of amphicarpy should provide additional insight into this plant reproductive strategy.

**Table 3:** Five attributes of amphicarpic plant species shared by many other species with a dimorphic reproductive system, e.g. aerial cleistogamous, amphi‐basicarpic and heterocarpic species that are not amphicarpic

Attribute	Explanation	References
Flower dimorphism	Morphological differences between cross‐ and self‐pollinated morphs	Plitmann (1995); Koontz *et al*. (2017)
Mixed mating system	Open, cross‐pollinated chasmogamous *versus* closed, self‐pollinated cleistogamous flowers	Goodwillie *et al*. (2005); Oakley *et al*. (2007)
Fruit and seed dimorphism (heterocarpy)	Differences in fruit and seed size and morphology	Imbert (2002); Baskin & Baskin (2014)
Germination dimorphism	Germination differences between morphs	Weiss (1980); Zhang *et al*. (2015)
Dispersal dimorphism	Differences in mechanism and distance of dispersal between morphs	Barker (2005); Zhang *et al*. (2015)

Previous studies provided information on seed dormancy and germination of amphicarpic plants at the whole‐seed level, but little is known about sub‐seed physiology and biochemistry or molecular biology of plants with this reproductive strategy. Thus, it is necessary to combine additional physiological and genetic techniques (genomics) to explain the mechanisms underlying the ecological functions of amphicarpy. For example, plants of *Gymnarrhena micrantha* produce both aerial and subterranean fruits under short days in the field. However, only aerial fruits developed under short days in a greenhouse and only subterranean fruits under long days outdoors (Evenari & Gutterman, 1966). What are the physiological processes involved in the accumulation of different metabolic and structural materials during aerial and subterranean seed development? For example, are there also hormonal interactions between aerial and subterranean seeds that influence their anatomical structure and germination? Why are aerial seeds of *Amphicarpaea bracteata* and *A. edgeworthii* physically dormant and orthodox (desiccation tolerant) and subterranean seeds physiologically dormant and recalcitrant (desiccation intolerant)? To answer these questions, a combined study is needed on the germination behaviour of aerial and subterranean seeds that takes into consideration the anatomical, sub‐whole‐seed physiological and possible genetic differences between aerial and subterranean seeds.

Empirical data also are needed to help explore the selective pressures and ecological factors that favour the evolution of amphicarpy. Cheplick (1994) hypothesized that the southerly‐distributed (subtropical) rhizomatous perennial *Amphicarpum muhlenbergianum* might be ancestral to the northerly distributed annual *A. amphicarpon*. Genetic markers could be used to compare the two species, and comparative life‐history data could be obtained to help support (or refute) the hypothesis. Genetic and ecological comparisons between amphicarpic species and closely related non‐amphicarpic congeners might shed light on the types of selective factors that contribute to the evolution of amphicarpy.

In addition, given that aerial and subterranean seeds are formed in different environments (above‐ and belowground) and at different positions on maternal plants, is the cue for their development in the external environment and/or is it due to some internal factor such as location of the meristem on the plant or some more general condition of the maternal plant? Does the position of the meristem on the maternal plant have any effect on external cues? An Evo‐Devo approach (Cheplick, 2015) to amphicarpy may provide important clues into genetic, environmental and developmental controls on the phenotypic plasticity of this reproductive system.

Thus far, little is known about the environmental and genetic determinants of the amphicarpic life history. By estimating the contribution of genetics to life‐history variation, insight can be gained as to how natural selection has led to the evolution of amphicarpy, a life‐history adaptation that has clearly been successful for some species in some habitats. Quantitative genetics techniques can be useful for estimating the heritability of reproductive traits, determining the consequences of amphicarpy for ecological genetics, studying natural selection of phenotypic traits important to fitness and determining the microevolutionary consequences of phenotypic variation in amphicarpic traits for adaptation to a specific habitat.

A mixed mating system has been reported for several amphicarpic species (e.g. Schnee & Waller, 1986; Trapp & Hendrix, 1988; Cheplick & Quinn, 1988*a*; Zhang *et al*., 2005). However, the extent to which aerial CH flowers of CL species actually outcross is not known. Also, almost all references to amphicarpy state that subterranean seeds are formed by self‐pollination of CL flowers, but there is no experimental evidence that this is the case. Thus, do subterranean flowers produce seeds by selfing or by parthenogenesis, apogamy or apospory? In addition, population genetic structure of amphicarpic *sensu lato* species is strongly affected by inbreeding *via* CL flowers (Cheplick, 2007; Swift *et al*., 2016). Although within‐population molecular genetic diversity may be low, high levels of genetic differentiation can occur between populations (Zhang *et al*., 2005; Liang *et al*., 2009). Common garden studies can reveal genetic differentiation (or its absence) among populations for phenotypic traits, whereas reciprocal transplant experiments can be used to document adaptation to local habitat conditions (Cheplick, 2015).

Investigations also would benefit greatly if genes responsible for the production of aerial and subterranean flowers in different species could be identified. Various techniques are now available, such as *in situ* hybridization to detect differences in gene expression patterns in aerial and subterranean seeds, real‐time reverse transcription‐polymerase chain reaction (RT‐PCR) analysis of gene expression in aerial and subterranean seeds, and isolating amphicarpic mutants by γ‐ray radiation, that can be used to help in gene identification. In addition, detailed micromorphological characterization and molecular analysis (comparative analysis of transcription and genome levels) of mutants need to be undertaken to elucidate the molecular characteristics of amphicarpy.

It seems likely that additional species with amphicarpy will be discovered in the future. As more amphicarpic species are identified, they can be used to test evolutionary hypotheses about the origin of amphicarpy and phylogenetic relationships among amphicarpic taxa. For example, there are five species in *Amphicarpaea*. *Amphicarpaea africana* (tropical Africa) and *A. ferruginea* (endemic to China) are perennial, and *A. bracteata* (North America), *A. edgeworthii* (E. Asia) and *A. linearis* (endemic to China) are annual. *Amphicarpaea africana*, *A. bracteata*, and *A. edgeworthii* produce aerial CH and CL flowers and subterranean CL flowers, whereas *A. linearis* and *A. ferruginea* produce only aerial CH flowers (Turner & Fearing, 1964; Zhang *et al*., 2015). Molecular genetic and phylogenetic research make it possible to estimate the approximate divergence times and number of times amphicarpy has evolved by comparing amphicarpic species with non‐amphicarpic congeners.

Most research on amphicarpy has been carried out on annual species. Thus, information is needed on the life history of perennial species with regard to trade‐offs between traits such as clonal reproduction *versus* sexual reproduction, CH *versus* CL breeding systems, and seed mass *versus* seed number of the two morphs. Comparisons of closely related amphicarpic species that differ in life form (e.g. annual *Amphicarpum amphicarpon versus* perennial *A. muhlenbergianum*) and possibly habitat requirements would provide insight into why the amphicarpic lifestyle evolved and how it may trade off with clonal (vegetative) growth.

Most amphicarpic plants, especially legumes, are underexploited or underutilized in their agriculture context. Legumes serve as a cheap source of protein for human and animals, and due to their ability to fix atmospheric nitrogen, they contribute to soil improvement (Tadele, 2014). There are 31 amphicarpic legumes. However, due to lack of genetic improvement, most amphicarpic plants are less productive in terms of both quality and quantity than domesticated legumes and would benefit from basic research. To boost productivity and diversify the food system, amphicarpic plants should be given due attention. Major improvements by conventional breeding technologies, such as selection of high‐yielding strains, hybridization and use of modern techniques developed for major crops, such as genome resequencing, selective sweep mapping, marker‐assisted selection (MAS) of desired allele(s) and allele combination(s), and genome‐editing technologies [such as clustered regularly interspaced short palindromic repeats/CRISPR‐associated protein 9 (CRISPR/CAS9)], also need to be applied to amphicarpic plants (Lenser & Theißen, 2013). The application of these techniques to amphicarpic plants would not only improve the quality of local diets but could also provide farmers with additional income as the seeds attract a market price well above the average for similar legumes (Tadele, 2009).

Furthermore, 67 species are reported to be amphicarpic, but the ecological significance of the production of aerial and subterranean diaspores has been studied for only a few of them. Whether the conclusions about amphicarpy based on the few taxa that have been studied can be generalized is open to question, and further studies on more species are needed.

## XII. CONCLUSIONS

1. Amphicarpy, the production of aerial and subterranean fruits/seeds on the same plant, maximizes fitness by combining the advantages of two reproductive strategies and is considered to be a bet‐hedging strategy.
2. Amphicarpy occurs in at least 67 herbaceous species belonging to 39 genera and 13 families of angiosperms that are phylogenetically widespread, suggesting that this life‐history strategy has evolved multiple times. Forty‐six per cent of the 67 known amphicarpic species are in the Fabaceae.
3. Amphicarpic species are less common in arid regions than in temperate and subtropical regions. In some temperate regions, amphicarpy may be an adaptation to escape fire.
4. Seeds from aerial and subterranean fruits differ in size, mass and/or degree of dormancy, dispersal mechanism and distance and ability to form a persistent seed bank, with aerial seeds generally being smaller, more dormant and more likely to be dispersed and to form a (persistent) seed bank than subterranean seeds. Furthermore, plants from subterranean seeds generally are larger, and seedlings are more tolerant of competition and stress and produce more aerial and subterranean seeds than those from aerial seeds.
5. Under limited resources (stress), plants produce subterranean seeds first and then shift to production of aerial seeds if there are sufficient resources. Thus, reproduction is assured (i.e. self‐pollination of subterranean CL flowers), and later in the growing season there may be increased reproductive output *via* aerial flowers.
6. Relatively little is known about the sub‐seed physiology of amphicarpic species, including biochemistry and molecular biology. Studies on genetics and proteomics would contribute to a better understanding of the adaptive ecological functions of amphicarpy and the selective forces favouring the evolution of this life‐history strategy.

## Supporting information


**Table S1.** Taxonomy, life form and geographical distribution of amphicarpic and amphi‐basicarpic species.
