# Supplementary material for: Amphicarpic plants: definition, ecology, geographic distribution, systematics, life history, evolution and use in agriculture
Source: Biol Rev Camb Philos Soc. 2020 May 28;95(5):1442–66. doi: 10.1111/brv.12623 (PMC7540684; doi:10.1111/brv.12623)
Supplement: Supplementary file 1 — Table S1. Taxonomy, life form and geographical distribution of amphicarpic and amphi‐basicarpic species. [file BRV-95-1442-s001.docx]

**Table S1.** Taxonomy, life form and geographical distribution of amphicarpic and amphi-basicarpic species. Information on geographical distribution was checked from the Kew database (<http://powo.science.kew.org/>) and Mabberley (2008). Therophytes are annuals, and all other life forms are perennials.

| **Family** | **Species** | **Life form** | **Distribution** | **References** |
| --- | --- | --- | --- | --- |
| **AMPHICARPY** | | | | |
| Asteraceae | *Catananche lutea* | Therophyte | Mediterranean region | Plitmann (1973); Cheplick (1987); Ruiz de Clavijo (1995); Ruiz de Clavijo & Jimenez (1998); Kaul *et al*. (2000); Lev-Yadun (2000) |
| Asteraceae | *Gymnarrhena micrantha* | Therophyte | N. Africa to C Asia & Pakistan, Arabian Peninsula | Koller & Roth (1964); Plitmann (1973); Zeide (1978); Cheplick (1987); Kaul *et al*. (2000); Lev-Yadun (2000) |
| Brassicaceae | *Cardamine chenopodifolia* | Therophyte | S U.S.A. | Gorczyński (1930); Cheplick (1983, 1987); Gamm (1983); Kaul *et al*. (2000) |
| Brassicaceae | *Geococcus pusillus* | Therophyte | S & SE Australia to Tasmania | Cheplick (1987); Kaul *et al*. (2000) |
| Commelinaceae | *Commelina benghalensis* | Geophyte | Tropical & S Africa, Madagascar, Arabian Peninsula | Maheshwari & Maheshwari (1955); Walker & Evenson (1985*a*,*b*); Cheplick (1987); Kaul *et al*. (2000, 2002) |
| Commelinaceae | *Commelina forskalaei* | Geophyte | Africa, SE Asia | Maheshwari & Maheshwari (1955); Cheplick (1987); Kaul *et al*. (2000); Barker (2005) |
| Commelinaceae | *Commelina indehiscens* | Chamaephyte | S India, Sri Lanka | Kaul *et al*. (2000) |
| Commelinaceae | *Commelina virginica* | Hemicryptophyte | C & E U.S.A. | Uphof (1938); Cardoso *et al*. (1994); Kaul *et al*. (2000) |
| Commelinaceae | *Murdannia nudiflora* | Therophyte | Tropical & subtropical Asia to W Pacific | Kaul *et al*. (2000), as *Commelina nudiflora* |
| Commelinaceae | *Tinantia pringlei* | Chamaephyte | NE Mexico | Parks (1935), as *Commelinantia pringlei* |
| Cyperaceae | *Eleocharis caespitosissima* | Therophyte or hemicryptophyte | Madagascar, N Australia | Bruhl (1994) |
| Fabaceae | *Amphicarpaea africana* | Therophyte | Tropical Africa | Barker (2005) |
| Fabaceae | *Amphicarpaea bracteata* | Therophyte | SE Canada, C & E U.S.A., Mexico | Schively (1897), as *Amphicarpaea monoica*; Allard (1932), as *Falcata comosa*; Schnee & Waller (1986); Cheplick (1987); Trapp (1988); Trapp & Hendrix (1988); Kaul *et al*. (2000), as *Amphicarpa monoica* |
| Fabaceae | *Amphicarpaea edgeworthii* | Therophyte | Himalaya to Japan | Fukui & Takahashi (1975); Zhang *et al*. (2005, 2006); Liang *et* *al*. (2009); Zhang *et al*. (2015) |
| Fabaceae | *Centrosema bracteosum* | Geophyte | E Bolivia to Brazil | Schultze-Kraft *et al*. (1997) |
| Fabaceae | *Centrosema grazielae* | Geophyte | Colombia to S Venezuela & Brazil | Schultze-Kraft *et al*. (1997) |
| Fabaceae | *Centrosema rotundifolium* | Geophyte | E Bolivia to Brazil | Schultze-Kraft *et al*. (1997), as *Centrosema heptaphyllum*; Kumar *et al*. (2012) |
| Fabaceae | *Centrosema venosum* | Geophyte | Colombia, Venezuela, Brazil | Schultze-Kraft *et al*. (1997) |
| Fabaceae | *Clitoria* sp.^a^ | ? | Brazil? | Schultze-Kraft *et al*. (1997) |
| Fabaceae | *Flemingia pauciﬂora* | Chamaephyte | N Australia | Kumar *et al*. (2012) |
| Fabaceae | *Galactia canescens* | Geophyte | Texas (U.S.A.) | Cheplick (1987); Kaul *et al*. (2000) |
| Fabaceae | *Glycine falcata* | Chamaephyte | C Australia | Doyle *et al*. (1990); Tindale & Craven (1988); Pfeil & Craven (2002); Kumar *et al*. (2012) |
| Fabaceae | *Glycine albicans* | Chamaephyte | NW Australia. | Doyle *et al*. (1990); Pfeil & Craven (2002) |
| Fabaceae | *Glycine arenaria^b^* | Chamaephyte | NW Australia to N Northern Territory, Australia | Tindale & Craven (1988); Pfeil & Craven (2002) |
| Fabaceae | *Glycine hirticaulis^b^* | Chamaephyte | N Northern Territory, Australia | Doyle *et al*. (1990); Tindale & Craven (1988); Pfeil & Craven (2002) |
| Fabaceae | *Glycine pindanica^b^* | Chamaephyte | NW Australia | Pfeil & Craven (2002) |
| Fabaceae | *Glycine pullenii^b^* | Chamaephyte | NW Australia to N Northern Territory, Australia | Pfeil & Craven (2002) |
| Fabaceae | *Glycine tomentella^b^* | Chamaephyte | China, Philippines, New Guinea, Australia, New Caledonia | Tindale & Craven (1988); Pfeil & Craven (2002) |
| Fabaceae | *Lathyrus amphicarpos* | Therophyte | Mediterranean region | Mattatia (1977*b*); Cheplick (1987); Kaul *et al*. (2000) |
| Fabaceae | *Lathyrus ciliolatus* | Therophyte | E Mediterranean region | Mattatia (1977*b*); Cheplick (1987); Christiansen *et al*. (1996); Kaul *et al*. (2000); Lev-Yadun (2000) |
| Fabaceae | *Lathyrus hierosolymitanus* | Therophyte | Aegean Islands to E Mediterranean region | Lev-Yadun (2000), as *Lathyrus hierosolymitanus* var. *amphicarpos* |
| Fabaceae | *Lathyrus hirticarpus* | Therophyte | Israel | Lev-Yadun (2000) |
| Fabaceae | *Lathyrus setifolius* | Therophyte | Mediterranean region | Mattatia (1977*b*); Cheplick (1987); Kaul *et al*. (2000), all as *Lathyrus setifolius* var. *amphicarpos* |
| Fabaceae | *Macroptilium gibbosifolium* | Hemicryptophyte | Arizona to Texas & C U.S.A., Cuba. | Schultze-Kraft *et al*. (1997), as *Macroptilium heterophyllum* |
| Fabaceae | *Macroptilium gracile*^c^ | Hemicryptophyte | SW Mexico to Tropical America | Pengelly & Eagles (1995); Schultze-Kraft *et al*. (1997) |
| Fabaceae | *Macroptilium panduratum* | Hemicryptophyte | Argentina, Brazil, Paraguay | Schultze-Kraft *et al*. (1997) |
| Fabaceae | *Macroptilium pedatum* | Hemicryptophyte | Mexico | Schultze-Kraft *et al*. (1997) |
| Fabaceae | *Neocracca* sp.^a^ | ? | S America | Schultze-Kraft *et al*. (1997) |
| Fabaceae | *Orobus* sp.^a^ | ? | N temperate zone | Schultze-Kraft *et al*. (1997) |
| Fabaceae | *Pisum fulvum*^d^ | Therophyte | Mediterranean region | Mattatia (1977*a*); Cheplick (1987); Kaul *et al*. (2000), as *Pisum fulvum* var. *amphicarpum*; Lev-Yadun (2000); |
| Fabaceae | *Tephrosia lupinifolia* | Therophyte or hemicryptophyte | Tropical Africa | Cheplick (1987); Kaul *et al*. (2000); Barker (2005) |
| Fabaceae | *Trifolium polymorphum* | Hemicryptophyte | Peru to S Brazil & S U.S.A. | Cheplick (1987); Kaul *et al*. (2000); Real *et al*. (2007); Conterato *et al*. (2010, 2013), all as *Trifolium argentinense*; Speroni *et al*. (2014) |
| Fabaceae | *Vicia angustifolia* | Therophyte | Macaronesia, temperate Eurasia, N Africa to Kenya | Alinoglu & Durlu (1970); Durlu & Cornelius (1970); Plitmann (1973); Cheplick (1987); Christiansen *et al*. (1996); Kaul *et al*. (2000); Lev-Yadun (2000), as *Vicia sativa* subsp. *amphicarpa* |
| Fabaceae | *Vicia sativa* var. *platysperma* | Therophyte | Temperate Eurasia to Arabian Peninsula | Barker (2005), as *Vicia amphicarpa* |
| Fabaceae | *Vigna lanceolata*^e^ | Therophyte or hemicryptophyte | Australia | White (1918); Dunlop & Webb (1991); Kumar *et al*. (2012) |
| Fabaceae | *Vigna minima* | Therophyte | China to Japan, Malesia to New Guinea | Gopinathan & Babu (1986, 1987); Cheplick (1987); Kaul *et al*. (2000) |
| Fabaceae | *Vigna radiata* | Therophyte | Arabian Peninsula, Taiwan to tropical Asia & NE Australia | Cheplick (1987); Kaul *et al*. (2000), as *Phaseolus sublobatus* |
| Gentianaceae | *Exochaenium oliganthum* | Therophyte, achlorophyllous | W tropical Africa to Ethiopia and Angola | Raynal (1967), as *Sebaea oligantha*; Kissling (2012); Merckx (2013) |
| Hydrocharitaceae | *Blyxa aubertii* | Hydrotherophyte or hydrohemicryptophyte | Tanzania to Mozambique, Madagascar, tropical and subtropical Asia to NW Pacific | Jiang & Kadono (2001) |
| Hydrocharitaceae | *Blyxa aubertii* var. *echinosperma* | Hydrotherophyte or hydrohemicryptophyte | Tropical and subtropical Asia to N Australia | Jiang & Kadono (2001), as *Blyxa echinosperma* |
| Hydrocharitaceae | *Ottelia ovalifolia* | Hydrohemicryptophyte | Australia | Ernst-Schwarzenbach (1956) |
| Poaceae | *Amphicarpum amphicarpon* | Therophyte | E U.S.A. | McNamara & Quinn (1977); Cheplick & Quinn (1982, 1983, 1987, 1988*b*); Cheplick (1987, 1988, 1989); Kaul *et al*. (2000), all as *Amphicarpum purshii* |
| Poaceae | *Amphicarpum muhlenbergianum* | Hemicryptophyte | SE U.S.A. | Cheplick (1987, 1994); Kaul *et al*. (2000), as *Amphicarpum floridanum* |
| Poaceae | *Chloris chloridea*^f^ | Hemicryptophyte | Arizona, Texas to C U.S.A. | Cheplick (1987); Kaul *et al*. (2000) |
| Poaceae | *Ent*e*ropogon chlorideus*^f^ | Hemicryptophyte | Arizona, Texas to C U.S.A. | Connor (1979); Cheplick (1987); Kaul *et al*. (2000), all as *Chloris chloridea* |
| Poaceae | *Eremitis* (*ca*, 5 species)^f^ | ? | E Brazil | Soderstrom & Calderón (1974); Cheplick (1987); Kaul *et al*. (2000), all as *Eremetis* |
| Poaceae | *Libyella cyrenaica*^f^ | Therophyte | NE Libya | Barker (2005) |
| Poaceae | *Paspalum amphicarpum* | Therophyte | Cuba | Connor (1979); Cheplick (1987); Kaul *et al*. (2000) |
| Polygalaceae | *Polygala pauciflora* | Therophyte | Cape Province, S Africa | Shaw (1904); Holm (1929); Koontz *et al*. (2017) |
| Polygalaceae | *Polygala polygama* | Chamaephyte | North America | Shaw (1904); Cheplick (1987); Kaul *et al*. (2000); Koontz *et al*. (2017) |
| Polygalaceae | *Polygala lewtonii* | Chamaephyte | Endemic to Florida (USA) | Swift *et* *al*. (2016) |
| Polygonaceae | *Emex australis* | Hemicryptophyte | Africa, Asia, Australia | Barker (2005) |
| Polygonaceae | *Emex spinosa* | Therophyte | Australia, Mediterranean countries | Plitmann (1973); Evenari *et al*. (1977); Weiss (1980); Cheplick (1987); Kaul *et al*. (2000); Lev-Yadun (2000); Ortiz *et* *al*. (2009); Sadeh *et* *al*. (2009); Berjano *et* *al*. (2014) |
| Polygonaceae | *Polygonum thunbergii* | Therophyte | NE Turkey to Caucasus, Russian Far East to N Indo-China & temperate E Asia | Kawano *et* *al*. (1990); Kaul *et al*. (2000); Kawano (2008); Choo *et al*. (2014, 2015); Kim *et al*. (2016); Nam *et al*. (2017), as *Persicaria thunbergii* |
| Polygonaceae | *Rumex bucephalophorus*^g^ | Therophyte | Mediterranean region | Talavera *et al*. (2010, 2011, 2012) |
| Scrophulariaceae | *Scrophularia arguta* | Therophyte | Macaronesia, S Spain, N & NE tropical Africa, Arabian Peninsula | Cheplick (1987); Kaul *et al*. (2000) |
| Urticaceae | *Laportea ovalifolia* | Chamaephyte | Tropical Africa | Engler (1895), as *Fleurya podocarpa*; Cheplick (1987); Kaul *et al*. (2000), as *Fleurya podocarpa* var. *amphicarpa*; Barker (2005) |
| Violaceae | *Viola tuberifera* | Therophyte | Himalaya to W & C China. | Gong *et al*. (2009) |
| **AMPHI-BASICARPY** | | | | |
| Amaranthaceae | *Ceratocarpus arenarius* | Therophyte | NE Bulgaria to Mongolia & Pakistan | Lu *et al*. (2014) |
| Cyperaceae | *Bulbostylis densa* | Therophyte | Tropical & subtropical Old World to S Russian Far East | Bruhl (1994) |
| Cyperaceae | *Bulbostylis glaberrima* | Therophyte | E Uganda to WC Kenya | Haines (1971); Bruhl (1994); Barker (2005) |
| Cyperaceae | *Bulbostylis humilis* | Therophyte | Yemen, Mozambique to S Africa | Haines (1971); Bruhl (1994); Barker (2005) |
| Cyperaceae | *Bulbostylis* sp. | Therophyte | E Africa | Haines (1971); Barker (2005) |
| Cyperaceae | *Bulbostylis sphaerocarpa* | Therophyte | E Sudan to Ethiopia, SW Tanzania | Haines & Lye (1983); Bruhl (1994) |
| Cyperaceae | *Bulbostylis stiratella* | Therophyte | E Africa | Haines & Lye (1983); Bruhl (1994) |
| Cyperaceae | *Schoenoplectiella aberrans* | Hemicryptophyte or rhizomatous geophyte | C Madagascar | Haines (1971); Barker (2005) as *Scripus aberrans* |
| Cyperaceae | *Schoenoplectiella articulata* | Therophyte | Tropical & subtropical Old World | Haines (1971); Barker (2005), as *Scripus articulata* |
| Cyperaceae | *Schoenoplectiella blackei* | Therophyte | Australia | Hayasaka (2009) |
| Cyperaceae | *Schoenoplectiella dissachantha^h^* | Therophyte | Australia | Lye (2003); Hayasaka (2012) |
| Cyperaceae | *Schoenoplectiella erecta* | Therophyte | Assam to Indo-China, Iberian Peninsula, Sahel to S Africa, Mascarenes, SE U.S.A., Texas and Mexico | Gordon-Gray *et al*. (2009), as *Schoenoplectus erectus* |
| Cyperaceae | *Schoenoplectiella hallii* | Therophyte | N C & E U.S.A. | Lye (2003); Smith *et al*. (2006); Hayasaka (2012) |
| Cyperaceae | *Schoenoplectiella heterophylla^h^* | ? | Madagascar | Lye (2003); Hayasaka (2012) |
| Cyperaceae | *Schoenoplectiella juncea^h^* | Therophyte | Ghana, Togo, Sudan, Somalia | Lye (2003); Hayasaka (2012) |
| Cyperaceae | *Schoenoplectiella laevis^h^* | Therophyte | Australia | Lye (2003); Hayasaka (2012) |
| Cyperaceae | *Schoenoplectiella lateriflora* | Therophyte or hemicryptophyte | Tropical & subtropical Old World to N Australia | Haines (1971); Barker (2005), as *Scripus lateriflora* |
| Cyperaceae | *Schoenoplectiella leucantha^h^* | Therophyte | S Africa | Lye (2003) |
| Cyperaceae | *Schoenoplectiella microglumis* | Therophyte | Ethiopia to Zambia | Lye (2003); Hayasaka (2012) |
| Cyperaceae | *Schoenoplectiella naikiana* | Therophyte | India | Hayasaka (2012) |
| Cyperaceae | *Schoenoplectiella oxyjulos* | Therophyte | W tropical Africa to S Sudan | Lye (2003); Browning & van der Burgt (2012) |
| Cyperaceae | *Schoenoplectiella patentiglumis^h^* | ? | W & S tropical Africa to KwaZulu-Natal | Hayasaka (2012) |
| Cyperaceae | *Schoenoplectiella perrieri* | Hemicryptophyte | W Madagascar | Haines (1971); Barker (2005), as *Scripus perrieri* |
| Cyperaceae | *Schoenoplectiella praelongata* | Therophyte | Tropical Asia to N Australia | Haines (1971); Barker (2005), as *Scripus praelongata* |
| Cyperaceae | *Schoenoplectiella raynaliana* | Therophyte | Togo, Chad to Ethiopia, S Africa, SW Arabian Peninsula | Lye (2003); Hayasaka (2012) |
| Cyperaceae | *Schoenoplectiella reducta* | Hemicryptophyte | SW Madagascar | Haines (1971); Barker (2005), as *Scripus reducta* |
| Cyperaceae | *Schoenoplectiella roylei^h^* | Therophyte | Tropical and S Africa, Iran to Indian Subcontinent | Lye (2003); Hayasaka (2012) |
| Cyperaceae | *Schoenoplectiella saximontana* | Therophyte | W Canada to W & C U.S.A., NE Mexico | Hayasaka (2012); Shiels *et al*. (2014) |
| Cyperaceae | *Schoenoplectiella senegalensis* | Therophyte or hemicryptophyte | Africa, S India | Lye (2003); Hayasaka (2012) |
| Cyperaceae | *Schoenoplectiella supinus* | Therophyte | Africa, Brazil to NE Argentina, Europe to Himalaya | Haines (1971); Barker (2005), as *Scripus supinus* |
| Cyperaceae | *Schoenoplectiella vohemarensis^h^* | ? | Madagascar | Lye (2003); Hayasaka (2012) |
| Cyperaceae | *Schoenoplectus muricinux* | Hemicryptophyte | Rwanda to S Africa | Haines (1971); Barker (2005), as *Scripus muricinux* |
| Cyperaceae | *Trianoptiles capensis* | Hemicryptophyte or rhizomatous geophyte | Cape Province, S Africa | Barker (2005) |
| Cyperaceae | *Trianoptiles solitaria* | Hemicryptophyte or rhizomatous geophyte | Cape Province, S Africa | Haines & Lye (1983); Barker (2005); de Roo (2002) |
| Cyperaceae | *Trianoptiles stipitata* | Hemicryptophyte or rhizomatouse geophyte | Cape Province, S Africa | Barker (2005) |
| Poaceae | *Enneapogon desvauxii* | Therophyte or Hemicryptophyte | Africa; temperate Asia to Indian Subcontinent; West-central U.S.A. to Mexico, Peru to Argentina | Barker (2005) |

^a^ Schultze-Kraft *et al*. (1997) list this genus as being amphicarpic, and Kumar *et al*. (2012), Conterato *et al*. (2013) and Lawn & Bielig (2016) cited this reference. However, we could not identify which species is (are) amphicarpic.

^b^ Species with ‘opportunistic’ amphicarpy were not counted in the main text.

^c^ In an analysis of 53 diverse accessions of the tropical forage legume *Macroptilium gracile* collected in Central and South America, Pengelly & Eagles (1995) noted that all but one of the amphicarpic accessions were collected from regions with mean annual rainfall < 1100 mm, whereas the non-amphicarpic forms generally came from wetter areas.

^d^ In *Pisum fulvum* (Fabaceae), there is a gradient from amphicarpic plants (*sensu stricto*) with both aerial and subterranean flowers and fruits to plants that produce only aerial flowers and fruits (Mattatia, 1977*a*). One of the stages in the gradient is a basicarpic form that produces chasmogamous flowers near the soil surface, which Mattatia (1977*a*) called ‘sub-amphicarpic’.

^e^ *Vigna lanceolata* consists of seven morphotypes, and amphicarpy has been observed in six morphotypes, among which two are annual, ﬁbrous-rooted forms and four rhizomatous perennial, tuberous-rooted forms (Lawn & Bielig, 2016). The four amphicarpic perennials are habitual. Of the two annuals, one exhibited habitual amphicarpy, whereas the other was opportunistic.

^f^ We could not find information about these species. We were unsure which species of *Eremitis* are amphicarpic, but counted *Eremitis* as five species in the main text.

^g^ In some populations of the heterocarpic *Rumex bucephalophorus* complex (Polygonaceae), plants that form a basal rosette may produce female flowers and mature diaspores in the leaf axils that are buried by the action of contractile roots (buried diaspores) as well as aerial hermaphrodite flowers and diaspores. On the other hand, plants that do not form a rosette produce only aerial flowers and diaspores. Altogether, one buried and three aerial diaspore types are produced by this species complex (Talavera *et* *al*., 2010, 2011, 2012).

^h^ The presence of amphi-basicarpy is the primary morphological trait Lye (2003) used to unite *Schoenoplectiella*, and all *Schoenoplectiella* section *Schoenoplectiella* taxa have been reported to exhibit amphi-basicarpy, if only rarely (Hayasaka, 2012). However, we could not find information about whether these species are amphi-basicarpic.
